# The short-chain fatty acid crotonate reduces invasive growth and immune escape of *Candida albicans* by regulating hyphal gene expression

**DOI:** 10.1128/mbio.02605-23

**Published:** 2023-11-06

**Authors:** Christopher McCrory, Jiyoti Verma, Timothy M. Tucey, Rachael Turner, Harshini Weerasinghe, Traude H. Beilharz, Ana Traven

**Affiliations:** 1 Department of Biochemistry and Molecular Biology and Infection Program, Biomedicine Discovery Institute, Monash University, Clayton, Australia; 2 Centre to Impact AMR, Monash University, Clayton, Australia; 3 Department of Biochemistry and Molecular Biology and Stem Cells and Development Program, Biomedicine Discovery Institute, Monash University, Clayton, Australia; The University of Texas Health Science Center, Houston, Texas, USA

**Keywords:** *Candida albicans*, gene expression, morphogenesis, macrophages, host-pathogen interactions, hyphal development, short chain fatty acids, crotonate, butyrate

## Abstract

Microbes are exposed to nutritional and stress challenges in their environmental and host niches. To rise to these challenges, they regulate transcriptional programs that enable cellular adaptation. For instance, metabolite concentrations regulate post-translational modifications of chromatin, such as histone acetylation. In this way, metabolic signals are integrated with transcription. Over the last decade, several histone acylations have been discovered, including histone crotonylation. Their roles in microbial biology, environmental adaptation, and microbe-host interactions are incompletely defined. Here we show that the short-chain fatty acid crotonate, which is used to study histone crotonylation, changes cell morphology and immune interactions of *Candida albicans*. Crotonate reduces invasive hyphal morphogenesis of *C. albicans* within macrophages, thereby delaying macrophage killing and pathogen escape, as well as reducing inflammatory cytokine maturation. Crotonate’s ability to reduce hyphal growth is environmentally contingent and pronounced within macrophages. Moreover, crotonate is a stronger hyphal inhibitor than butyrate under the conditions that we tested. Crotonate causes increased histone crotonylation in *C. albicans* under hyphal growth conditions and reduces transcription of hyphae-induced genes in a manner that involves the Nrg1 repressor pathway. Increasing histone acetylation by histone deacetylase inhibition partially rescues hyphal growth and gene transcription in the presence of crotonate. These results indicate that histone crotonylation might compete with acetylation in the regulation of hyphal morphogenesis. Based on our findings, we propose that diverse acylations of histones (and likely also non-histone proteins) enable *C. albicans* to respond to environmental signals, which in turn regulate its cell morphology and host-pathogen interactions.

**IMPORTANCE:**

Macrophages curtail the proliferation of the pathogen *Candida albicans* within human body niches. Within macrophages, *C. albicans* adapts its metabolism and switches to invasive hyphal morphology. These adaptations enable fungal growth and immune escape by triggering macrophage lysis. Transcriptional programs regulate these metabolic and morphogenetic adaptations. Here we studied the roles of chromatin in these processes and implicate lysine crotonylation, a histone mark regulated by metabolism, in hyphal morphogenesis and macrophage interactions by *C. albicans*. We show that the short-chain fatty acid crotonate increases histone crotonylation, reduces hyphal formation within macrophages, and slows macrophage lysis and immune escape of *C. albicans*. Crotonate represses hyphal gene expression, and we propose that *C. albicans* uses diverse acylation marks to regulate its cell morphology in host environments. Hyphal formation is a virulence property of *C. albicans*. Therefore, a further importance of our study stems from identifying crotonate as a hyphal inhibitor.

## INTRODUCTION

Changes to chromatin structure are fundamental to the regulation of gene expression in eukaryotes. One of the important ways in which chromatin is modified is by reversible acetylation of lysines in histones (1). This serves to either activate or repress transcription, depending on the cell’s needs.

Acetylation is the most abundant and best studied histone acylation, but other acyl groups can also be reversibly attached to histones (2–7). Regardless of their lower abundance, these non-acetyl acylations (e.g., crotonyl, propionyl, lactyl, and succinyl) are thought to have relevant roles in gene expression because of their ability to regulate transcription, as well as evidence that supports their links with transcriptional control in development, in relation to cellular metabolism, immunometabolic shifts, and in response to DNA damage and stress (3, 4, 8–13). Cellular metabolic pathways generate the donors for these histone marks, i.e., their respective acyl-coAs (14). Acyl-coA concentrations dictate the stochiometry of acylations on chromatin (15) and can change in response to metabolic signals, including those induced by diets, nutrition, and supplementation of metabolites such as short-chain fatty acids (SCFAs) (3, 4, 13, 16). These metabolites work on chromatin in a couple of ways: (i) they are metabolized to produce the acyl-coA donors for histone acylations (3, 13), and (ii) some, such as butyrate, are inhibitors of histone deacetylases (HDACs) (17) and can thereby increase several histone acylations (13, 18). Since SCFAs are produced by bacteria, including those residing in the gut (19), the non-standard acylations can regulate host-microbe interactions. For example, butyrate produced by gut bacteria increases histone crotonylation in colon epithelial cells (18). Butyrate also reduces hypoxia-induced brain damage by regulating crotonylation of histone and non-histone proteins (20). However, much is still to be learned about the roles of the non-acetyl acylations in host-microbe interactions.

To improve our understanding of this question, we are focusing on the yeast *Candida albicans. C. albicans* lives in the human gastrointestinal tract and oral and vaginal microbiomes and is a frequent cause of opportunistic fungal infections (21). *C. albicans* responds to metabolic challenges in its human body niches (22). We thus hypothesize that there are opportunities for *C. albicans* to use multiple chromatin acylations to regulate its adaptive gene expression programs. In support of this hypothesis, we have shown that the levels of histone crotonylation in *C. albicans* vary upon stress and metabolic change (16). The crotonyl-lysine marks are recognized by the YEATS domain (23–25), which is present in several proteins within chromatin modification complexes and general transcription factors (26). We have shown that the *C. albicans* YEATS protein Taf14 is required for virulence in the mouse bloodstream infection model and is also involved in several phenotypes *in vitro*, such as stress responses and growth in hyphal morphology (16). We also found that, when *C. albicans* is grown in the presence of the SCFA crotonate, histone crotonylation increases and many genes are differentially expressed (16). Zhou et al. defined the *C. albicans* crotonylome, identifying over 5,200 crotonylation sites on 1,584 proteins with diverse functions (27). Collectively, these studies suggest that lysine crotonylation has a significant impact on *C. albicans* biology, but this impact is poorly defined.

The same histone lysine residues are modified by more than one acylation, and the histone acetyltransferases and deacetylases are also shared [reviewed in reference (1)]. This means that it is impossible to distinguish the biological roles of specific acylations by mutations in these factors. To circumvent this, a frequently used tool to study histone crotonylation is supplementation of crotonate, which makes chromatin more crotonylated in mammalian and fungal cells (12, 13, 16, 28). Here we took this approach to understand the roles of crotonylation in host-pathogen biology, focusing on *C. albicans*-macrophage interactions.

## RESULTS

### Crotonate changes the outcomes of *C. albicans-*macrophage interactions

Macrophages phagocytose *C. albicans* to control its growth within tissues and organs. Studies *in vitro* have shown that, within macrophages, *C. albicans* encounters nutritional and stress challenges but is able to adapt its metabolism to survive and grow (29). Further to this, *C. albicans* develops invasive hyphal morphology (30). Hyphae destroy macrophages by a number of mechanisms, leading to fungal release (31–34). During this process, hyphae break the macrophage phagosomal membrane, which activates the NLRP3 inflammasome (31, 35, 36). As such, hyphal morphogenesis within macrophages is involved in both immune escape by *C. albicans* and in immune recognition that leads to anti-microbial inflammation.

**Fig 1:**
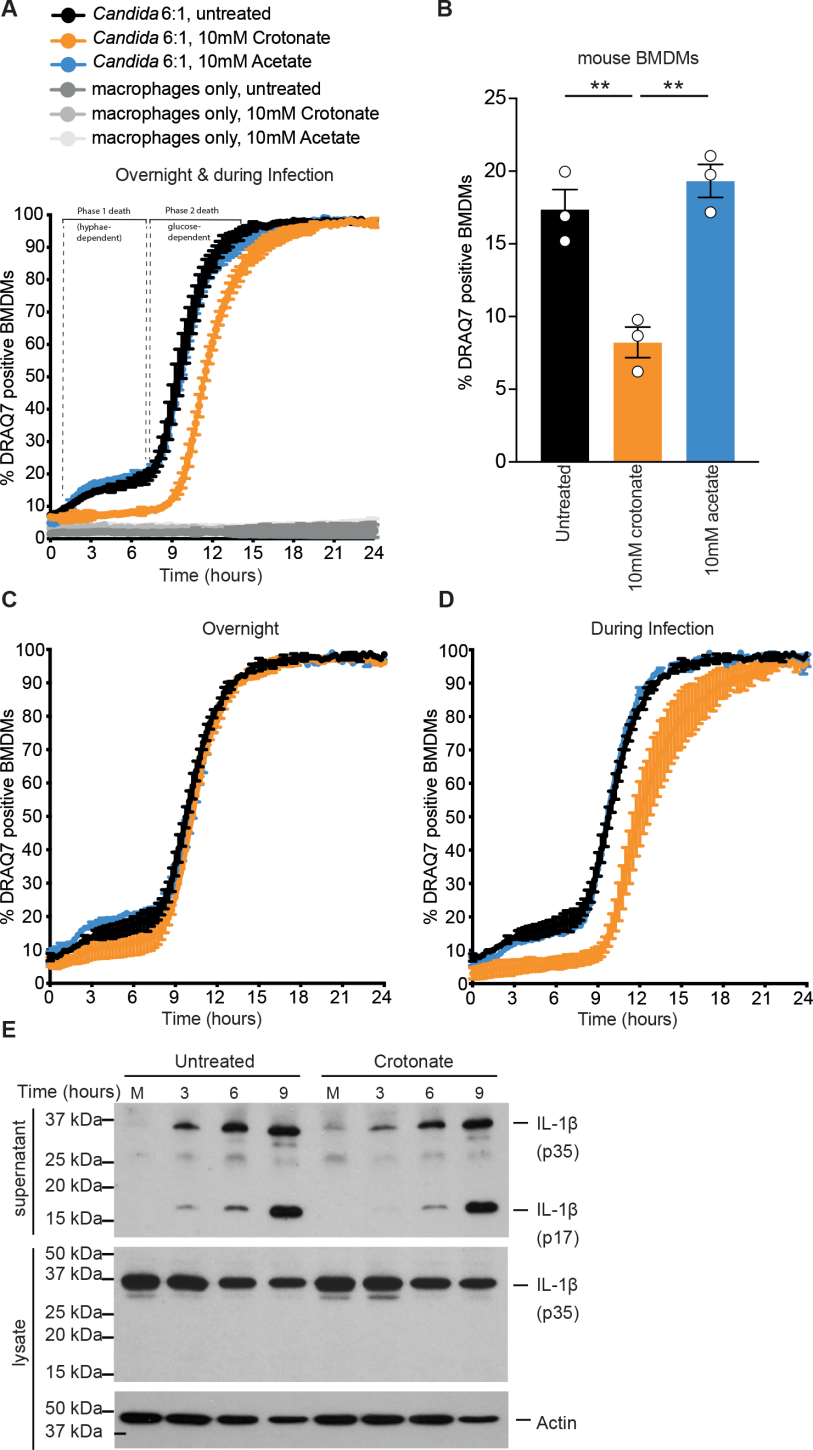
Crotonate modulates macrophage viability and inflammatory cytokines upon fungal challenge. (**A**) Quantification of macrophage (BMDMs) cell death by live cell imaging following infection with *C. albicans*. BMDMs were seeded overnight with or without 10 mM crotonate or acetate followed by *Candida* infection at a multiplicity of infection (MOI) of 6. Crotonate or acetate remained present in the medium during the course of infection. Shown is the percentage of DRAQ7-positive BMDMs over time. Data are the mean values and standard error of the mean (SEM) from three independent experiments for all infected conditions, two replicates for uninfected macrophages ± crotonate and one replicate for uninfected macrophages + acetate. At least 2,000 macrophages were surveyed for each condition, per experiment. (**B**)Percentage DRAQ-7 positive BMDMs at 6 h post-infection from the experiments in panel A. Mean and SEM are shown. Statistical significance was determined by one-way analysis of variance followed by Tukey’s multiple comparisons test. **P < 0.005. (**C**) Live cell imaging quantification of macrophage cell death as in panel A. Crotonate or acetate were present only overnight (i.e., pre-phagocytosis of *Candida*). Data are the mean values and SEM from two independent experiments and at least 3,000 macrophages surveyed for each condition, per experiment. These experiments were done together with two of the three experiments presented in panel A. (**D**) Live cell imaging quantification of macrophage cell death as in panel A. Crotonate or acetate was present only during infection (post-phagocytosis). Data are the mean values and SEM from two independent experiments and at least 3,000 macrophages surveyed for each condition per experiment. These experiments were done together with two of the three experiments presented in panel A. (**E**) Western blot analysis of interleukin (IL)-1β following infection with *C. albicans* with or without 10 mM crotonate, and supernatants and lysates collected at the indicated time points. Crotonate caused reduced cleavage of IL-1β in the first 3–6 h after infection. Shown is one representative immunoblot from three independent experiments. Uncropped Western blots from all three independent experiments are shown in Fig. S3.

We used crotonate as a tool to study the roles of lysine crotonylation during *C. albicans* infection of macrophages. In our system with bone marrow-derived mouse macrophages (BMDMs), hyphae-dependent macrophage lysis occurs soon after infection and lasts for approximately 9 h (Fig. 1A) and (32). After escape, *C. albicans* further kills macrophages by depleting glucose via extracellular hyphal growth (37) (Fig. 1A). Crotonate significantly improved the viability of macrophages in the first 9 h after challenge (Fig. 1A and B). We have previously shown that acetate, a related SCFA, has no effect on macrophage cell death (37), and we could reproduce that result here (Fig. 1A and B). The glucose-dependent death of macrophages was not reduced by crotonate (Fig. 1A). Since *Candida*’s ability to deplete glucose depends on its proliferation, these results suggest that crotonate is not working simply by inhibiting fungal growth. Indeed, crotonate’s effect on *C. albicans* growth was minimal when assayed *in vitro* in macrophage infection medium (Fig. S1A and B).

In *C. albicans*, the increase in histone crotonylation due to crotonate is rapidly reversed upon crotonate’s removal (16). The same dynamic regulation occurs in macrophages: crotonate increased macrophage histone crotonylation, and this was reduced within 30 min of its removal (Fig. S2). In the experiments in Fig. 1A, we pre-treated macrophages with crotonate and also kept crotonate present in the medium during infection. In parallel experiments, we show that macrophage rescue is seen only when crotonate is present during infection, but not if it is removed after the pre-treatment (Fig. 1C and D). This is consistent with the effects of crotonate in the macrophage experiments being driven by increased lysine crotonylation, which requires the continued presence of crotonate.

Hyphae-dependent macrophage death is driven in part by NLRP3 inflammasome-dependent pyroptosis (31, 32). Reduced macrophage cell death (shown in Fig. 1A) is consistent with reduced pyroptosis, suggesting lower NLRP3-dependent responses in the presence of crotonate. Consistently, crotonate reduced NLRP3/caspase-1 dependent cleavage of the proinflammatory cytokine interleukin (IL)-1β at 3 and 6 h post-infection (Fig. 1E; Fig. S3).

### Reprogramming of the *C. albicans* transcriptome by crotonate

In the hope of explaining how crotonate reduces macrophage cell death and proinflammatory activation, we used RNAseq to understand its effects on the transcriptional programs in macrophages and/or *Candida*. We infected macrophages with *C. albicans* and analyzed transcriptomes after 1 and 3 h, comparing samples with crotonate to those without. These two time points were chosen to (i) assess the early response to crotonate while *C. albicans* is still intracellular in macrophages at 1 h and (ii) understand how it adapts to crotonate over time at 3 h when escape is evident, especially in control conditions. The entire data set can be viewed and analyzed at https://rnasystems.erc.monash.edu/~retur3/AS5_39/degust/

The macrophage data varied between the three biological repeats (repeat 3 was clearly different from the other two) (Fig. S4A). Nevertheless, our overall conclusion is that crotonate did not trigger major changes in the macrophage transcriptome. We make this conclusion because no differentially expressed genes were found using false discovery rate (FDR) 0.05 and 1.5-fold change at either one or 3 h post-infection, with or without repeat 3 included in the analysis. This is also shown by the clustering of samples by time point rather than crotonate treatment (Fig. S4B and C).

**Fig 2:**
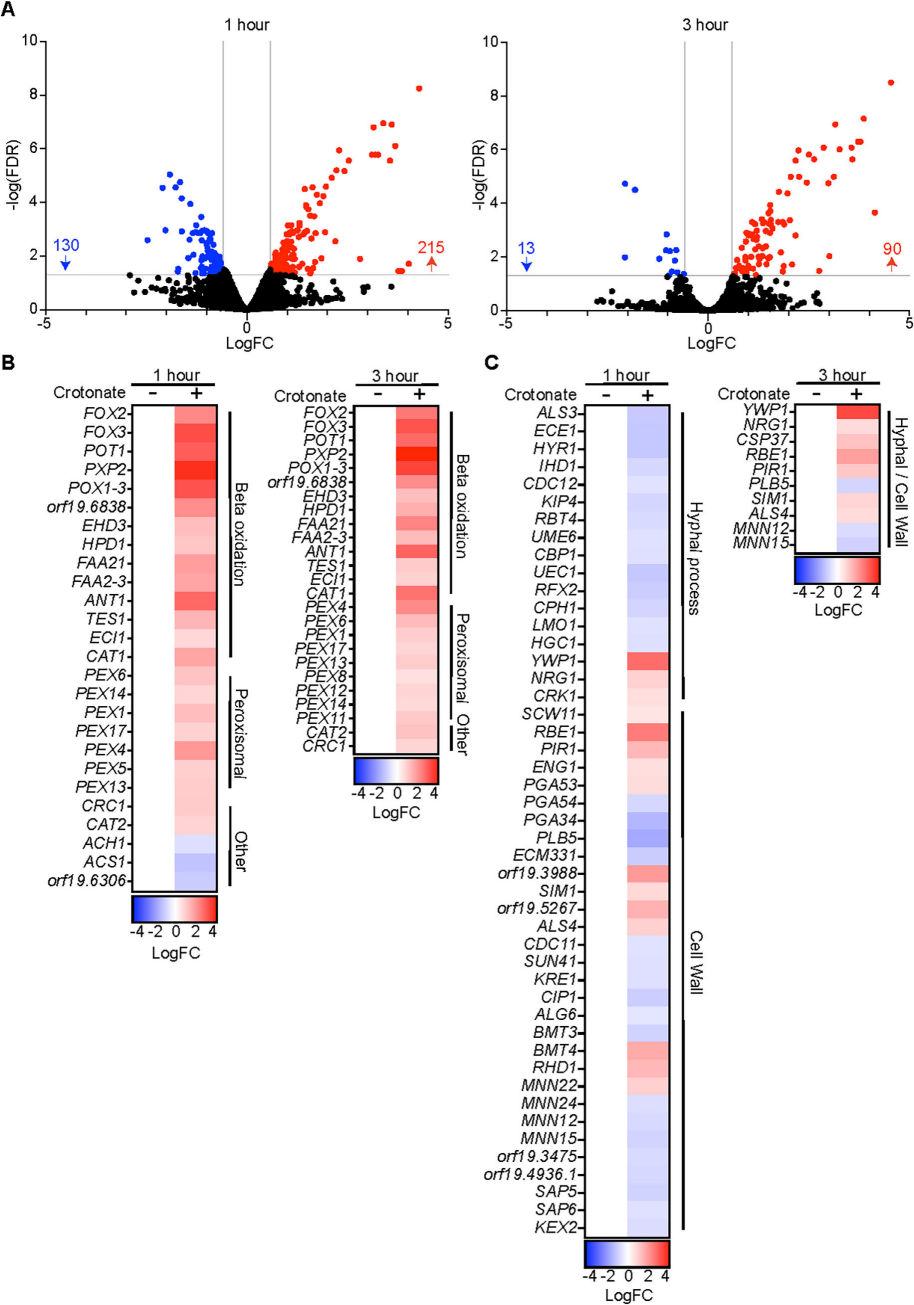
Crotonate reprograms the *C. albicans* transcriptome during macrophage infections. (**A**) Volcano plot of *C. albicans* differentially expressed gene at 1 and 3 h in the presence of crotonate. (**B**) Heat map of β-oxidation and associated genes functions. Genes were assigned to these biological roles based on knowledge from *C. albicans* (candidagenome.org) or the functions of their *S. cerevisiae* homologs. (**C**) Heat map of hyphal and cell wall biogenesis genes. The differentially expressed core hyphal response genes determined in the Kadosh lab study (39) are included.

The transcriptional response to crotonate was stronger for *C. albicans*, with 345 and 103 differentially expressed genes at 1 and 3 h, respectively (FDR 0.05, 1.5-fold change) (Fig. 2A; Data set S1). A smaller response at 3 h indicates that *C. albicans* adapts to crotonate over time and/or crotonate’s effects are reduced as fungal escape initiates. A pronounced biological response of *C. albicans* to crotonate was upregulation of the β-oxidation pathway and peroxisome biogenesis (Fig. 2B; gene ontology analysis is in Data set S1). Crotonate also upregulated several associated functions, such as the peroxisomal catalase *CAT1* involved in detoxification of hydrogen peroxide formed during β-oxidation, two mitochondrial enzymes (*EHD3* and *HPD1*) that participate in a modified β-oxidation pathway involved in the breakdown of propionyl-coA to acetyl-coA (38), and the carnitine acyltransferases *CAT2* and *CRC1* that are required for shuttling acyl-coA metabolites between the peroxisome and mitochondria (Fig. 2B; Data set S1). The acetyl-coA synthetase *ACS1* and the acetyl-coA hydrolase *ACH1*, which are involved in acetyl-coA formation from acetate and vice versa, were downregulated by crotonate, as was the carnitine biosynthesis enzyme encoded by orf19.6306 (CR_04,870C) (Fig. 2B). Consistent with some effects on cell growth, crotonate upregulated the enzymes involved in *de novo* purine biosynthesis and genes related to ribosome biogenesis and rRNA and ncRNA processes, although the majority of these genes were upregulated only mildly (between 1.5 and 2.0-fold), and their upregulation was seen at the 1 h time point but not at 3 h (Data set S1).

**Fig 3:**
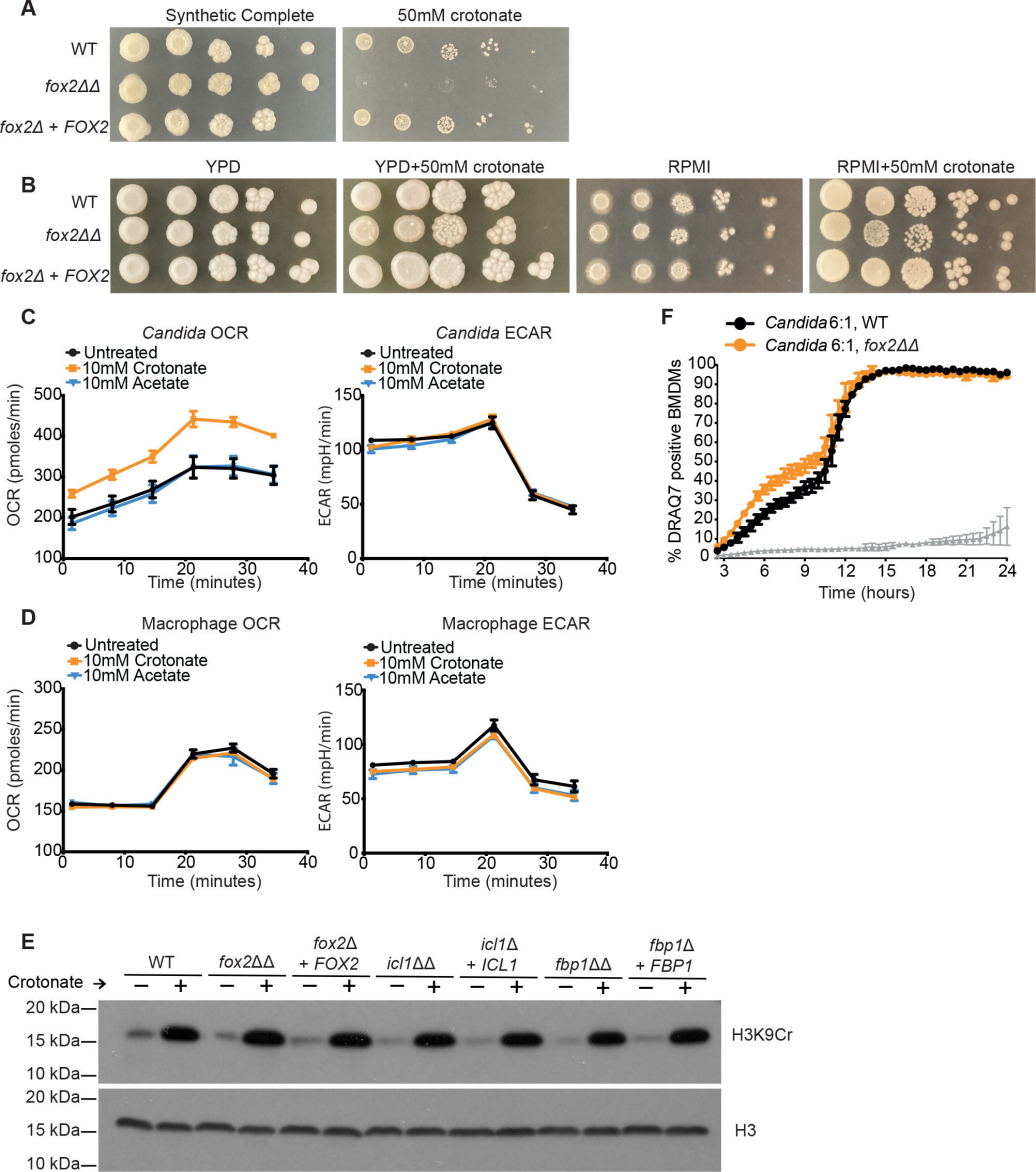
β-oxidation is required for crotonate utilization by *C. albicans* but not for histone crotonylation during standard laboratory growth. (**A**) Growth of *fox2*Δ/Δ, complemented strain and wild-type (WT) *C. albicans* on 50 mM crotonate as the sole carbon source. Plates were photographed after 3 days of growth at 30°C. (**B**) Growth of *fox2*Δ/Δ, complement and wild type on RPMI and YPD (yeast extract, peptone, dextrose) medium with or without 50 mM crotonate. Plates were photographed after 3 days of growth at 30°C. (**C**) Seahorse analysis showing oxygen consumption rate (OCR) and extracellular acidification rate (ECAR) levels as measured from *Candida* cells. Cells were treated with or without 10 mM crotonate or acetate for 1 h before as well as during Seahorse measurements. Data are the mean values and SEM from two independent experiments; each independent experiment involved two different colonies treated as biological replicates (therefore, *n* = 4). (**D**) Seahorse analysis showing OCR and ECAR in BMDMs. Cells were treated with or without 10 mM crotonate or acetate for 1 h before as well as during Seahorse measurements. Data are the mean values and SEM from two independent experiments. (**E**) Western blots for crotonylated H3K9 (H3K9cr) and H3 as loading control for the indicated strains, grown in YPD at 30°C with or without 50 mM crotonate. Shown is one representative immunoblot from two independent experiments. Uncropped Western blots from both experiments are shown in Fig. S5A. (**F**) Live cell imaging of macrophage viability over time following infection with the wild type or *fox2*Δ/Δ mutant of *C. albicans* (MOI 6). Shown is the percentage of DRAQ7-positive macrophages (BMDMs) over time from two independent experiments (average and SEM). At least 2,000 macrophages were surveyed for each condition per experiment. The gray line represents uninfected macrophages.

The upregulation of β-oxidation suggested that *C. albicans* might be metabolizing crotonate. Indeed, *C. albicans* could grow on crotonate as the sole carbon source and the β-oxidation pathway was required, as shown by lack of growth of the β-oxidation mutant *fox2*Δ/Δ (Fig. 3A). Both the wild type and the *fox2*Δ/Δ mutant grew normally in the presence of crotonate if glucose was present as a carbon source (Fig. 3B). This result shows that crotonate is not toxic to *C. albicans* even when it cannot be metabolized by the β-oxidation pathway. Consistent with effects on fungal metabolism, crotonate increased the oxygen consumption rate (OCR) in *C. albicans* (Fig. 3C). The extracellular acidification rate (ECAR), which is a measure of glycolysis, was not affected (Fig. 3C). Acetate had no effect on either OCR or ECAR (Fig. 3C). Crotonate did not change OCR or ECAR in macrophages (Fig. 3D), in line with our transcriptomics data showing minimal effects of crotonate on the host transcriptome (Fig. S4).

In *Saccharomyces cerevisiae*, the β-oxidation pathway has been implicated in the production of crotonyl-coA for histone crotonylation under growth conditions that cause cells to enter the yeast metabolic cycle (12). In *C. albicans*, the *fox2*Δ/Δ mutant displayed normal basal levels of crotonylated H3K9 (H3K9cr) under standard laboratory growth in rich medium and normal increases in response to crotonate (Fig. 3E; Fig. S5A). The glyoxylate cycle mutant *icl1*Δ/Δ and the gluconeogenesis mutant *fbp1*Δ/Δ also displayed normal levels of H3K9cr (Fig. 3E). Finally, the *fox2*Δ/Δ mutant killed macrophages normally (Fig. 3F). Collectively, these data show that while fungal metabolism is impacted by crotonate, this does not appear to regulate *C. albicans*-macrophage interactions.

### Crotonate impairs hyphal-specific gene transcription and hyphal morphogenesis

The transition of *C. albicans* from yeast to hyphal morphology is controlled by a transcriptional program, which is activated in response to environmental signals that lead to hyphal morphogenesis (40–42). The RNAseq data showed that crotonate caused differential expression of cell wall and hyphae-related genes in *C. albicans* during macrophage infections at the 1 h time point (Fig. 2C; Data set S1). Specifically, several genes known to be activated during hyphal morphogenesis were repressed by crotonate (Fig. 2C). These include 9 out of 15 genes of the core hyphal response (39), such as *ECE1*, *ALS3*, *HYR1*, *RBT4*, and others (Fig. 2C; Data set S1). In contrast, *YWP1* encoding a cell wall protein expressed in yeast but not hyphae was upregulated by crotonate (Fig. 2C). The transcriptional activators of hyphal genes *CPH1* and *UME6* were downregulated (although generally the change in expression was <2fold), while the hyphal repressor *NRG1* was upregulated (Fig. 2C). Another transcriptional repressor implicated in hyphal morphogenesis, *RFX2*, was downregulated (Fig. 2C; Data set S1). Overall, these transcriptional changes are consistent with reduced hyphal gene expression in response to crotonate at 1 h, while *C. albicans* is predominantly found inside macrophages. Reduced expression of hyphae-related genes in the presence of crotonate was lost by 3 h (Fig. 2C; Data set S1), suggesting that *C. albicans* adapts to crotonate over time.

**Fig 4:**
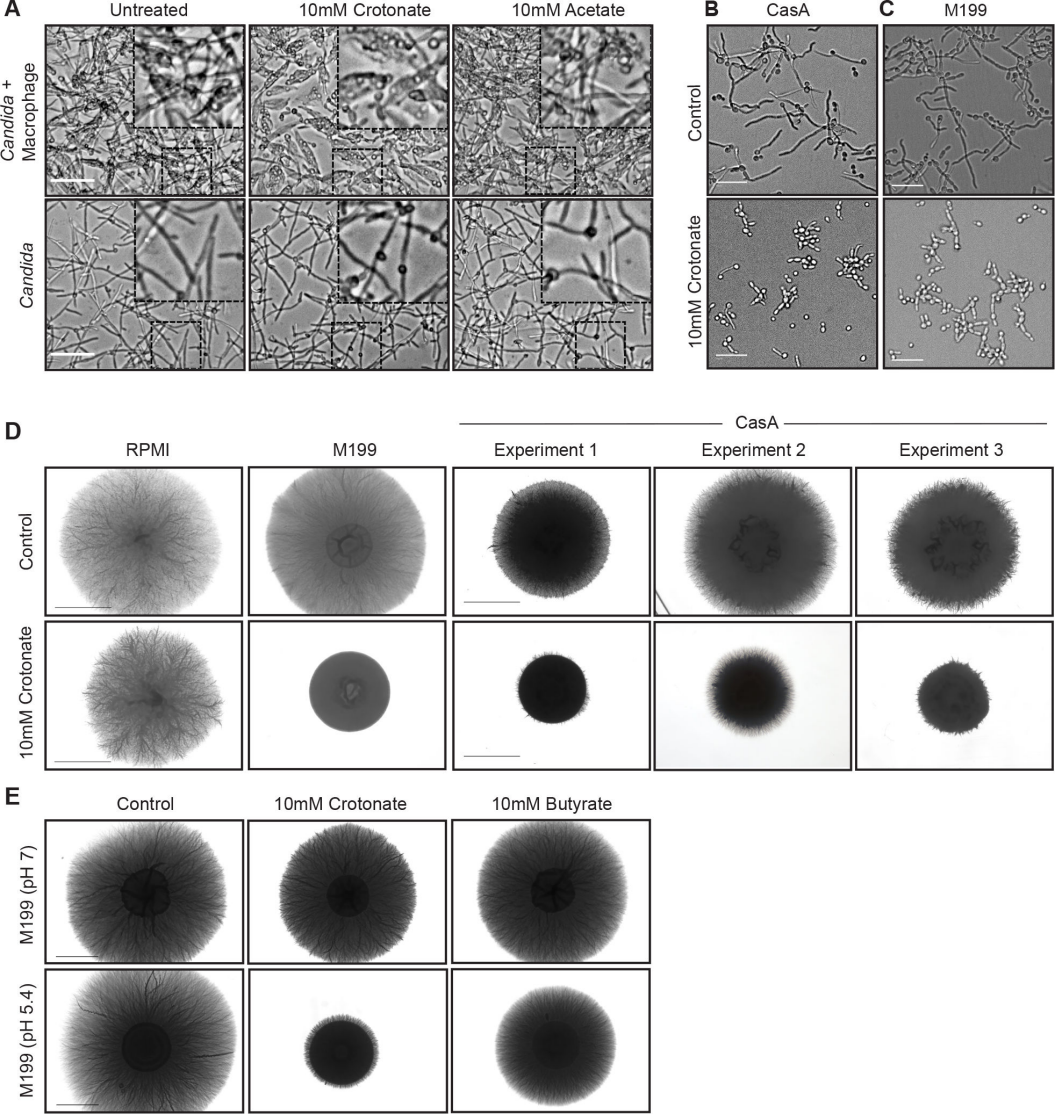
Crotonate represses hyphal morphogenesis. (**A**) Cell morphology of *C. albicans* in macrophages (BMDMs) 3 h post-phagocytosis. The infection was performed in the presence of absence of 10 mM crotonate or acetate as described in Fig. 1A (MOI 6). Scale bar is 50 µm. In parallel, *C. albicans* cell morphology was imaged during growth in macrophage tissue culture media (BMDM medium, which is RPMI based) but without macrophages. Scale bar is 50 µm. The right top corner shows magnified cells of the square shown in the image. For live cell imaging of hyphal growth in RPMI medium ± crotonate, see Movie S1. (**B**) Cell morphology of *C. albicans* in the presence or absence of crotonate in liquid CAS (casaminoacids) medium (assayed in tissue culture conditions at 37°C and 5% CO_2_). Scale bar is 50 µm. Also see Movie S2. (**C**) Cell morphology of *C. albicans* in the presence or absence of crotonate in liquid M199 medium (assayed in tissue culture conditions, 37°C and 5% CO_2_). Scale bar is 50 µm. Also see Movie S3. (**D**) Colony morphology of *C. albicans* on the indicated plates in the presence or absence of crotonate. Cells were plated and grown at 37°C for 7 days. Hyphal growth is seen as “hair” around the colonies. Scale bar is 4 mm. (**E**) Colony morphology of *C. albicans* in the presence or absence of crotonate or butyrate (both at 10 mM) grown on M199 plates at pH 7.0 or pH 5.4. The experiment was done as in panel D. Scale bar is 4 mm.

Lower transcription of hyphae-specific genes suggests impairment of hyphal morphogenesis by crotonate. This would explain how crotonate lowers macrophage cell death and IL-1β maturation, which are both promoted by hyphae (30–32, 36, 43). Indeed, crotonate reduced hyphal formation in macrophages and resulted in *C. albicans* remaining within macrophages for longer: at 3 h post-infection, abundant extracellular hyphae were visible in controls but not in the presence of crotonate (Fig. 4A). Consistent with metabolic effects of crotonate having a limited or no effect on *C. albicans* behavior within macrophages, the *fox2*Δ/Δ mutant formed normal hyphae within macrophages (Fig. S5B). Surprisingly however, although crotonate inhibited hyphal formation in macrophages, the same concentration of crotonate (10 mM) did not inhibit hyphal formation when *C. albicans* was grown on its own in macrophage infection medium (Fig. 4A) or RPMI + 10% fetal bovine serum (FBS) medium (Movie S1).

The macrophage infection medium is RPMI-based and as such differs from the macrophage phagosome in nutrient composition such as carbon sources (fatty acids and amino acids in the phagosome, glucose in RPMI) as well as pH (acidic in the phagosome, neutral in RPMI). Following phagocytosis of *C. albicans* yeast cells, the pH of macrophage phagosome was measured to be around 5 for a couple of hours, after which it increases concomitant with hyphal elongation (44). Mimicking the phagosomal environment with CAS medium, which contains casamino acids as the carbon source and has an acidic pH (we measured a pH of 5.9–6.0), caused hyphal inhibition by crotonate (Fig. 4B). *C. albicans* continued to grow in the presence of crotonate in CAS medium but as pseudohyphae (elongated cell chains) rather than the hyphal filaments seen in controls (Movie S2). A similar phenotype was observed in M199 medium which contains glucose and has a phagosome-mimicking pH of 5.4. Crotonate inhibited hyphal growth in M199 medium and caused the formation of pseudohyphae and cell chains (Fig. 4C; Movie S3). Growth curve measurements showed a moderately reduced growth in medium M199 in the presence of crotonate at 37°C, but no difference at 30°C (Fig. S1C and D). Cell proliferation in crotonate is also seen in Movie S2. Similar effects were seen on solid medium: crotonate’s repression of hyphal formation was not seen on RPMI plates but was evident on M199 and CAS plates (Fig. 4D). There was more variation in hyphal inhibition seen on CAS plates compared to M199, potentially explained by the slightly higher pH or the relative extent of hyphal filamentation in control conditions in the independent experiments (Fig. 4D shows some examples across the spectrum of phenotypes from independent experiments). Acetate did not inhibit hyphal growth on M199 medium (Fig. S6). Since both crotonate and acetate are used as sodium salts, we also show that NaCl at the same concentration shows no effect (Fig. S6).

RPMI and M199 both contain glucose as a carbon source but differ in pH (7.2 for RPMI and 5.4 for M199). We thus hypothesized that acidic pH, as is seen in the phagosome, was the key to promoting crotonate’s anti-hyphal activity. To test this, we raised the pH of M199 to pH 7.0 and compared it to pH 5.4. Inhibition of hyphae by crotonate was clearly evident in pH 5.4 but not in pH 7.0 (Fig. 4E). The better-studied SCFA butyrate was previously reported to inhibit hyphal formation by *C. albicans* (45–47). We could reproduce this effect, but butyrate was a weaker hyphal inhibitor than crotonate in our experiments using M199 plates (Fig. 4E). Similar to crotonate, hyphal inhibition by butyrate was also stronger in acidic pH (Fig. 4E). Collectively, these data show that crotonate and butyrate inhibit hyphal morphogenesis, depending on environmental conditions, with strong inhibition seen in conditions that recapitulate the macrophage phagosome.

### The Nrg1-dependent pathway is implicated in hyphal repression by crotonate

**Fig 5:**
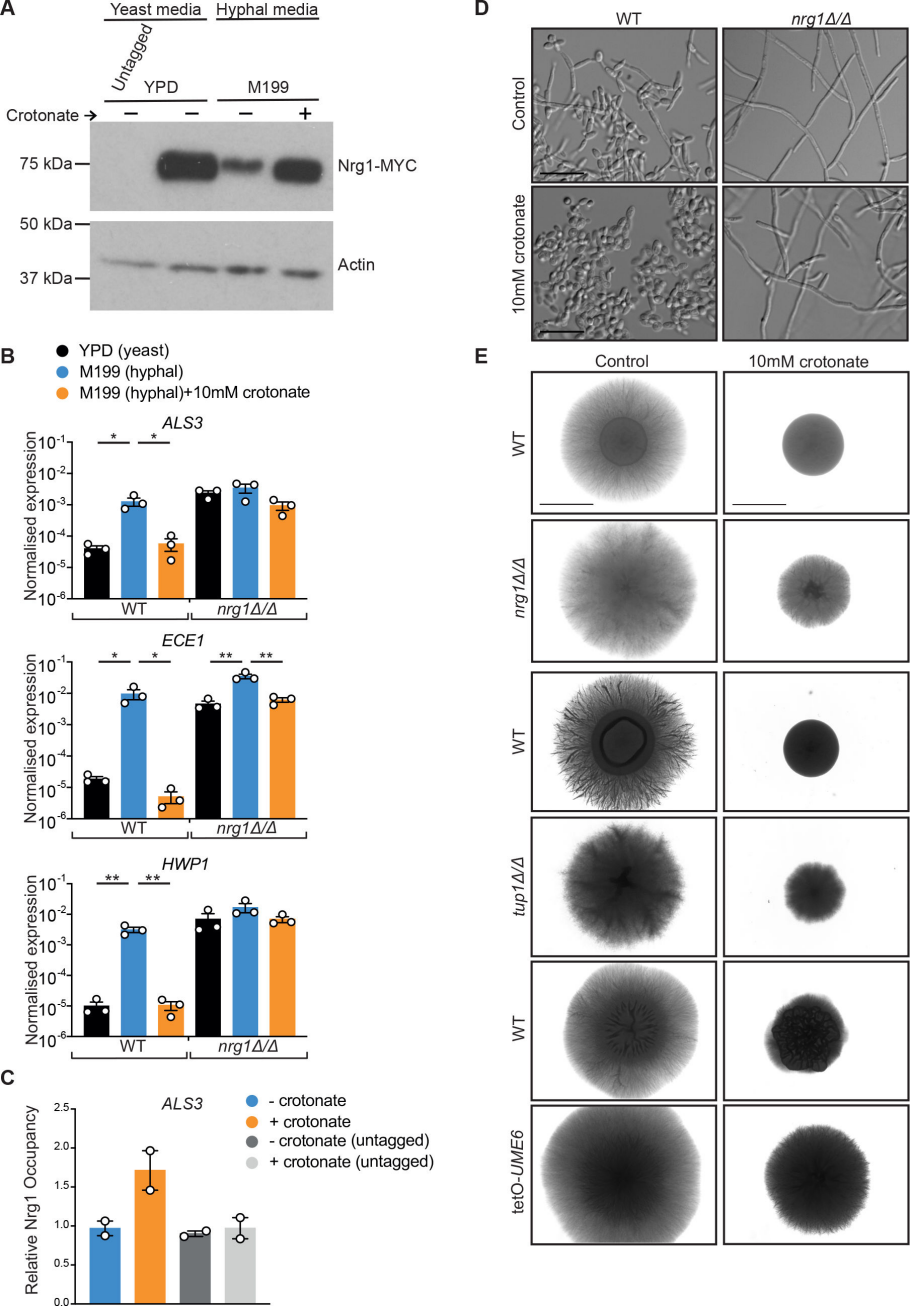
Crotonate regulates hyphal gene expression and morphogenesis via Nrg1. (**A**) Western blot of Nrg1-MYC± crotonate. Yeast morphology was maintained in YPD at 30°C, while hyphal conditions were M199 pH 5.4 at 37°C. Nrg1-MYC ran at a molecular size of 75 kDa, as we have seen before and determined this band to correspond to Nrg1 (52). Uncropped Westerns are shown in Fig. S7. (**B**) Quantitative PCR (qPCR) for the expression of the hyphal genes *ALS3*, *HWP1*, and *ECE1* in wild-type (WT) or *nrg1*Δ/Δ strains normalized to *RDN25*. Overnight cultures were diluted to OD_600_ of 0.2 and transferred to YPD at 30°C for control yeast morphology or to M199 medium for 5 h at 37°C to induce hyphal morphogenesis, in the presence or absence of crotonate as indicated. Shown are the average and SEM of three independent experiments. Statistics represent a one-way analysis of variance followed by multiple comparisons test. *P < 0.05, **P < 0.01. (**C**) Chromatin immunoprecipitation samples were grown in M199 ± 10 mM crotonate at 37°C for 3 h. DNA samples were quantified by qPCR with primers in the promoter region of *ALS3* between −424 and −290. The *ACT1* open reading frame was used as control. The Nrg1 occupancy is presented for both Nrg1-MYC and untagged control as fold enrichment over *ACT1* following calculation of percentage of input. Shown are the average and SEM of two biological repeats. (**D**) Hyphal morphogenesis of WT or *nrg1*Δ/Δ in liquid M199 medium (5 h of growth at 37°C). Scale bar is 20 µm. (**E**) Hyphal morphogenesis of the indicated strains on M199 plates (7 days of growth at 37°C). Scale bar is 4 mm. Mutants were analyzed in separate experiments, and the WT control is shown for each of them.

Hyphae-specific genes are repressed by the transcriptional repressor Nrg1 (40, 41, 48). As such, initiation of hyphal-specific transcription necessitates the reduction of Nrg1 protein levels to stop transcriptional repression (42, 48, 49). We therefore tested if hyphal repression by crotonate involved the Nrg1-pathway. In the presence of crotonate the mRNA levels of *NRG1* were increased during macrophage infections (Fig. 2C). Moreover, we observed increased Nrg1 protein levels in the presence of crotonate during growth in hyphae-inducing M199 medium (Fig. 5A; Fig. S7). In M199 medium, crotonate repressed the expression of the hyphal-induced genes *ECE1*, *ALS3*, and *HWP1* (Fig. 5B), and this repression was largely (although not completely) dependent on Nrg1: hyphal genes were derepressed in the *nrg1*Δ/Δ mutant, and their repression by crotonate was much reduced (Fig. 5B and C). By chromatin-immunoprecipitation (IP), we found an enrichment of Nrg1 at the promoter of the hyphal gene *ALS3* in the presence of crotonate, consistent with its involvement in hyphal repression by crotonate (Fig. 5C). Further, the constitutively hyphal growth of the *nrg1*Δ/Δ mutant was not inhibited by crotonate in either liquid or solid medium (Fig. 5D and E). We did, however, observe diminished growth of *nrg1*Δ/Δ colonies on plates in the presence of crotonate (Fig. 5E). This suggests that crotonate’s effects on long term growth on plates are mediated by additional, Nrg1-independent pathways. A similar effect of crotonate (reduction of growth but not filamentation) was seen for the *tup1*Δ/Δ mutant, which lacks the Tup1 co-repressor that works in concert with Nrg1 (Fig. 5E). One of the important ways in which Nrg1 regulates *C. albicans* morphology is by repressing *UME6*, a key transcriptional activator of hyphal genes (50). *UME6* transcript levels were reduced in crotonate-treated *C. albicans* in our RNAseq data set (Fig. 2B) and repression by crotonate was incomplete when *UME6* levels were increased using doxycycline-regulated expression (51) (Fig. 5E).

### The histone deacetylase inhibitor trichostatin A partially rescues hyphal repression by crotonate

**Fig 6:**
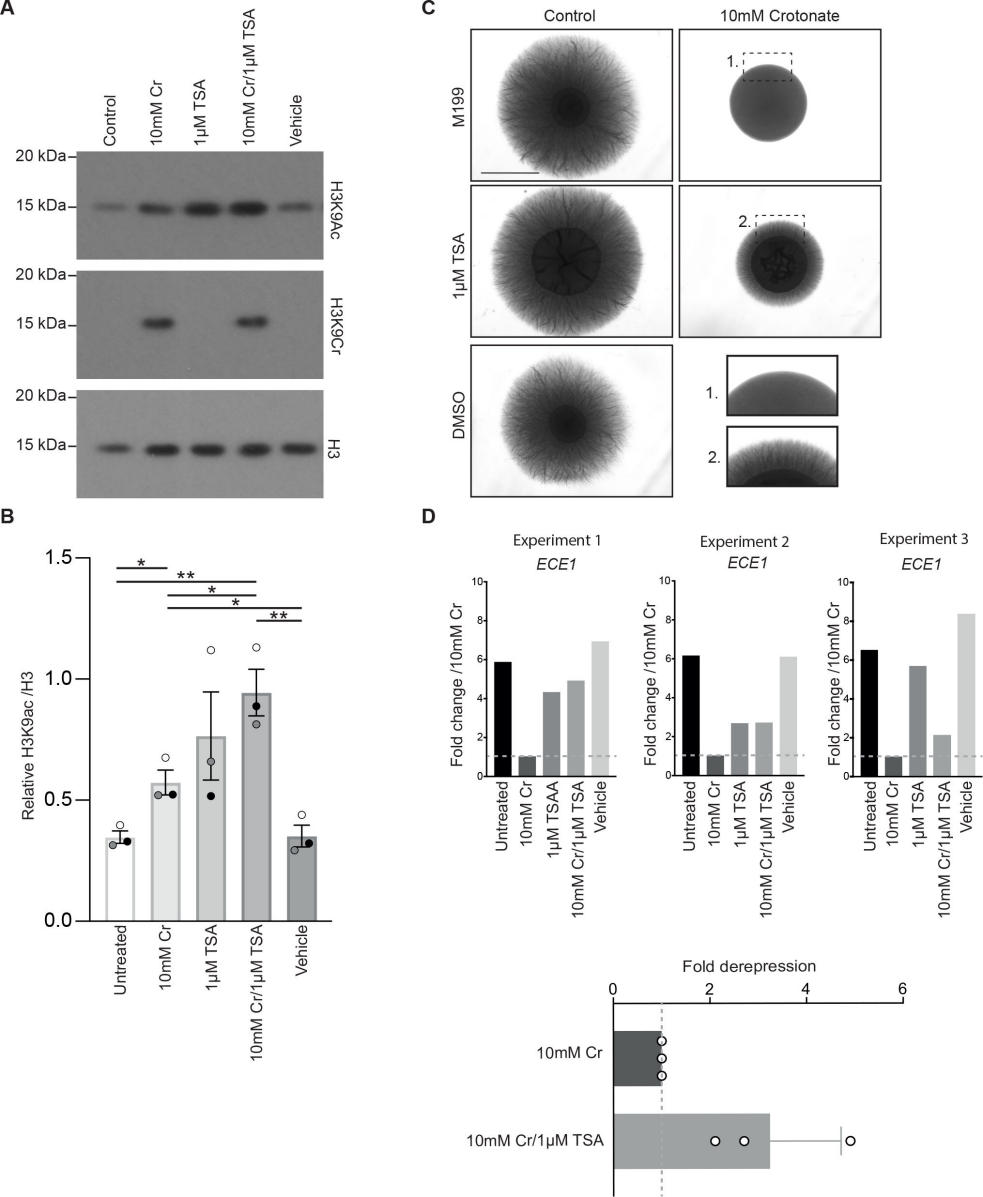
The HDAC inhibitor trichostatin A (TSA) partially rescues hyphal inhibition by crotonate. (**A**) Western blot analysis of H3K9cr, H3K9ac, and total H3 following growth of *C. albicans* in M199 medium for 2 h, in the presence or absence of crotonate (Cr) or TSA at the indicated concentrations. Three gels were loaded in parallel with the same samples, and membranes probed with antibodies recognizing crotonylated or acetylated H3K9 peptides or total H3 as indictated. To increase our ability to detect crotonylated H3, 2.5× protein extract was loaded into gels probed with the ani-H3K9cr antibody relative to the other two gels) Uncropped Western blots and three independent repeats are shown in Fig. S8. (**B**) Quantification of H3K9ac relative to total H3 from the experiment shown in A and two other independent experiments shown in Fig. S8. The three experiments are differentiated by the color of the data points (black, white, and gray). Statistics represent unpaired *t*-tests comparing each condition to every other separately. *P < 0.05, **P < 0.01. (**C**) Hyphal morphogenesis of wild-type *C. albicans* grown on M199 plates in the presence or absence of crotonate and TSA at the indicated concentrations, alone or in combination. Enlarged outcrops (1) and (2) allow for improved visualization of colony morphology. Images were taken after 7 days of growth at 37°C. Scale bar is 4 mm. (**D**) qPCR of hyphal gene expression from cells collected from M199 plates, in the presence or absence of crotonate and TSA at the indicated concentrations, alone or in combination. Shown are three independent experiments and the fold differences relative to the 10 mM crotonate condition, which was set to 1.

Chromatin regulation has been implicated in hyphal growth and the transcription of hyphal-specific genes, including their regulation by Nrg1 (49, 53–56). Supplementation of crotonate to *C. albicans* cultures growing in M199 medium resulted in a large increase in H3K9cr (Fig. 6A; Fig. S8A, antibody specificity controls are shown in Fig. S8C). Crotonate also increased acetylated H3K9 (H3K9ac) (Fig. 6A and B; Fig. S8B), which is consistent with some activity as an HDAC inhibitor (13). The increase in histone crotonylation in hyphal conditions parallels what we previously observed in response to crotonate in yeast conditions (16).

Given that the same lysine residues in histones (including H3K9) are modified by acetylation and crotonylation (1) (also see Fig. 6A and S8A), we wondered if addition of crotonate and the consequent increase in histone crotonylation could be competing with acetylation, thereby causing the observed reduction in hyphal growth. To test this, we asked if increasing histone acetylation by supplementation of the HDAC inhibitor trichostatin A (TSA) can rescue *C. albicans* from hyphal inhibition by crotonate. Excitingly, this was the case. In the TSA + crotonate samples, the levels of H3K9ac increased relative to crotonate alone in three independent experiments (Fig. 6A and B, S8A and S8B), and TSA partially rescued hyphal filamentation in the presence of crotonate (compare plates with crotonate and TSA relative to crotonate alone) (Fig. 6C). More examples of hyphal rescue by TSA are shown in Fig. S9, including some cases where we saw improved colony growth on crotonate + TSA plates relative to crotonate alone. TSA alone did not affect hyphal growth at the concentration of 1 µM (Fig. 6C), although we observed reduced expression of the hyphal-specific gene *ECE1* (Fig. 6D; compare “untreated” with TSA data). Of note, repression of *ECE1* by TSA was not as strong as the repression by crotonate (Fig. 6D). Moreover, the strong repression of *ECE1* caused by crotonate was partially rescued by TSA, with a two- to fourfold upregulation in the TSA + crotonate condition relative to crotonate alone (Fig. 6D). This is consistent with TSA’s ability to cause a partial rescue of hyphal morphogenesis in the presence of crotonate.

## DISCUSSION

Here we used the SCFA crotonate as a tool to address the roles of lysine crotonylation in microbe-host interactions. We found that crotonate reduces hyphal growth of *C. albicans* within macrophages. This confines *C. albicans* inside macrophages for longer, slows macrophage cell death, and reduces maturation of the inflammatory cytokine IL-1β. By these mechanisms, crotonate improves fungal control by macrophages and reduces inflammation that could be damaging to the host.

The donor for histone crotonylation, crotonyl-coA, is produced by metabolic processes in the cell (14). This could be its physiological source within *C. albicans* cells during macrophage infections. Indeed, we observe H3K9cr under default conditions (i.e., without crotonate addition; see reference 16 and Fig. 3C), which shows that *C. albicans* chromatin is crotonylated. Crotonyl-coA is also produced by metabolizing the SCFA crotonate (13). Supplementation of crotonate causes a large increase in histone crotonylation in *C. albicans* (see reference 16 and Fig. 6A), which is consistent with a substantial increase in the intracellular levels of crotonyl-coA in response to crotonate determined in mammalian cells (13). It will be interesting to determine how crotonate-induced crotonylation compares to physiological levels. Analyses of histone acylations in mammalian cells grown in standard tissue culture showed the relative abundance of crotonate to be 1%–3% (15). We can assume something similar for *C. albicans*; however, the abundance of histone crotonylation might change in response to the environment: carbon source availability might drive the intracellular metabolic pathways that create crotonyl-coA, or SCFAs in the gut might be metabolized to crotonyl-coA or act on chromatin directly via their HDAC activity to increase histone crotonylation, as has been shown for butyrate (18). To our knowledge, the concentrations of crotonate in the human gut are unknown. However, based on the metabolic capacity of bacteria found in the gut, it has been suggested that crotonate is present (57, 58).

We also addressed two other SCFAs produced by gut bacteria: acetate and butyrate. In our experiments, acetate had no effect on hyphal morphogenesis, fungal or macrophage metabolism, or *C. albicans*-induced macrophage cell death. Butyrate inhibited hyphal growth, which is line with previous reports (45–47), but it was a weaker hyphal inhibitor than crotonate in our conditions. The anti-hyphal activity of both crotonate and butyrate was heightened at acidic pH, which mimics macrophage phagosomes and gut environments. Acidic pH could increase the intracellular accumulation of SCFAs, since protonated SCFAs would be expected to cross the cell membrane more effectively. Overall, our results support anti-hyphal roles of SCFAs in host-mimicking conditions.

Infection of macrophages with pathogens, such as methicillin-resistant *Staphylococcus aureus* and the parasite *Toxoplasma gondii*, results in changes to crotonylation of the macrophage proteome with functional consequences (59, 60). In lipopolysaccharide (LPS)-activated macrophages (RAW247.6 cell line), addition of crotonate led to increased histone crotonylation and increased expression of several genes (13). It was thus surprising that, although crotonate induced histone crotonylation in *C. albicans*-infected macrophages, we detected minimal changes to macrophage transcriptomes and no change to macrophage oxidative or glycolytic metabolism. Differences in immune cell types (mouse BMDMs in our study versus human THP-1, mouse RAW247.6, and porcine alveolar macrophages in the other studies) are a possible explanation for these different results.

Our data suggest that crotonate acts on the *C. albicans*-macrophage interaction by affecting the pathogen. Crotonate induced metabolic changes in *C. albicans*, elevating transcription of the β-oxidation pathway and increasing the oxygen consumption rate (Fig. 2 and 3). Consistently, we could show that *C. albicans* metabolizes crotonate via the β-oxidation pathway. In this context, it is relevant that Zhou et al. identified lysine crotonylation on many *C. albicans* proteins with metabolic functions, including central metabolic enzymes (27). These crotonylations would be expected to increase when crotonate is supplemented to the medium, as is the case in our experiments. Crotonate’s metabolic effects could impair hyphal formation by *C. albicans*, but our analyses of the β-oxidation mutant *fox2*Δ/Δ did not reveal any changes to hyphal morphogenesis or ability to cause macrophage cell death. We also did not detect changes to H3K9 crotonylation in *fox2*Δ/Δ during growth in rich medium *in vitro*, although we acknowledge that ß-oxidation could have a role in the production of crotonyl-coA for histone crotonylation under nutrient-limiting conditions such as are found in host niches. In addition to the metabolic effects, we observed that crotonate caused repression of hyphal gene transcription, both during macrophage infection and in *C. albicans* growing *in vitro* in hyphae-inducing medium. We propose that this hyphal repression is, at least in part, the reason behind low hyphal morphogenesis in the presence of crotonate.

How does crotonate repress hyphal gene expression? Crotonate strongly increased the levels of crotonylated H3K9 in hyphal-inducing conditions (Fig. 6), providing a way in which it could regulate transcription. Lysine crotonylation is generally thought of as a histone mark that activates transcription (13). It was therefore surprising that our experiments showed that crotonate represses hyphal-specific genes. There are, however, precedents for transcriptional repression by histone crotonylation. For example, during the yeast metabolic cycle, histone crotonylation leads to transcriptional repression of growth-related genes (12), and we can envisage several mechanisms by which histone crotonylation could repress transcription of hyphal genes in *C. albicans*. Firstly, it is possible that repression is an indirect effect of activating a repressor. Indeed, our data are consistent with the Nrg1 repressor pathway being involved. Firstly, both protein and mRNA levels of Nrg1 were increased by crotonate, showing that crotonate interferes with downregulation of Nrg1 that is required for the transcription of hyphae-specific genes (48, 49). Furthermore, repression of the hyphal genes *ECE1, ALS3* and *HWP1* by crotonate was largely dependent on Nrg1. Nrg1 was enriched at promoters of *ALS3* in the presence of crotonate, and crotonate did not repress hyphal growth in the absence of Nrg1 or its co-repressor Tup1 (although colony size was reduced). At present, it is unclear if crotonate regulates the Nrg1 pathway directly or indirectly.

A second possibility is that there is competition between crotonylation and acetylation of histone lysine residues, since the same lysines are modified by these acylations. In support of this mechanism, we show that HDAC inhibition by TSA increases the levels of acetylated H3K9 in the presence of crotonate and partially restores hyphal growth. The competition between crotonylation and acetylation of histones could regulate chromatin binding of the *C. albican*s bromodomain protein Bdf1, since bromodomains interact with acetylated lysines, but have much lower affinity for crotonylated lysines (61). Crotonylation would therefore be expected to reduce the interaction of Bdf1 with chromatin, as has been shown in mammalian cells (62). Bdf1 is a subunit of the Swr1 chromatin remodeling complex, which has been implicated in the regulation of yeast to hyphal morphogenesis in *C. albicans* (63). However, Swr1 was located on hyphal-specific gene promoters in yeast cells but not hyphae (63), which complicates this explanation. It should also not be ignored that many non-histone proteins are acylated (27), and therefore, dynamic crotonylation/acetylation of non-histone proteins could also regulate hyphal morphogenesis, and possibly also the improvement in colony growth by TSA that we observed in some experiments (Fig. S9). Finally, the experimental system that we used here manipulates the levels of histone crotonylation and acetylation quite dramatically by supplementation of crotonate and TSA; thus, future experiments will need to address how crotonylation and acetylation might compete under physiological conditions to regulate fungal morphogenetic programs.

In summary, based on our findings, we propose that histone crotonylation regulates the hyphal transcriptional program in *C. albicans*, thereby impacting on hyphal growth and host-pathogen interactions. Dynamic switching of *C. albicans* between distinct morphologies plays roles in both commensal and pathogenic mechanisms (64). Our data suggest that *C. albicans* has the ability to use diverse histone acylations to control its yeast and hyphal morphologies in response to environmental and metabolic signals. This proposition fits with the known roles of chromatin in regulating hyphal gene transcription and the morphogenetic transition between yeast to hyphae in *C. albicans* (reviewed in references 65, 66). Given the importance of hyphae for the virulence of *C. albicans* (30; reviewed in reference 64), our identification of crotonate as an inhibitor of hyphal growth and macrophage escape shows that the morphogenetic effects of SCFAs (also shared by butyrate as shown here and by others [45–47]) could have implications for promoting commensalism of *C. albicans* over its pathogenesis.

## MATERIALS AND METHODS

### Yeast and macrophage growth conditions

The *C. albicans* strain SC5314 was used in the majority of experiments. Metabolic mutants of *C. albicans* were a gift from Mike Lorenz and are described in reference (67). The Nrg1-MYC strain was a gift from Haoping Liu and is described in reference 49. The *nrg1*Δ/Δ and *tup1*Δ/Δ mutants and their control strain SN250 are described in the Homann collection (68). We obtained the collection from the Fungal Genetics Stock Centre (69). The tetO*-UME6* strain and its tetR control were a gift from David Kadosh and are described in reference (51).

Yeast cultures were grown on YPD (1% yeast extract, 2% peptone, 2% glucose, and 2% agar) at 30°C. Hyphal growth was induced in Medium 199 (buffered with HEPES), pH 5.4; RPMI-1640, pH 7.2 (buffered with MOPS); or CAS, pH 5.9–6 (minimal medium with 0.67% yeast nitrogen base, containing all amino acids and 2% casamino acids). All media included 80 µg/mL uridine. RPMI and M199 were adjusted to the desired pH using NaOH. Medium for growth on crotonate as a sole carbon source was 0.67% yeast nitrogen base, containing all amino acids and crotonate. Crotonate, acetate, and butyrate were used as sodium salts and obtained by diluting concentrated acetic, butyric, and crotonic acid with ddH_2_O before adjusting to pH 7.4 and filter sterilizing.

Growth assays on plates were performed by using overnight fungal cultures serially diluted in 10-fold increments starting at the optical density (OD_600_) of 0.5. Growth was assessed after growth at 30°C for 3 days. For the growth curves in liquid medium, singles colonies were grown overnight at 30°C in YPD. Cells were washed and diluted to a starting OD_600_ of 0.2 into 200 µL BMDM media with or without 10 mM crotonate in a 96-well plate. Readings were done using the Tecan Spark plate reader at either 30°C or 37°C over 24 h with shaking (200 rpm). OD_600_ was read every 30 min. Growth curves in M199 were conducted manually using 10 mL of medium in flasks over a 24 h period and measuring OD_600_ at 2, 4, 6, 8, 16, 20, and 24 h.

Our protocols for the isolation and growth of murine BMDMs are described (37).

### Live cell imaging

Live cell imaging experiments were performed using our established protocols described in (37). BMDMs were seeded in either 24-well plates at 5 × 10^5^ cells/well or in 96-well plates at 1 × 10^5^ cells/well, with or without 10 mM crotonate or acetate as indicated in the figures. The multiplicity of infection (MOI) was 6. The live cell imaging procedure and analysis of data were performed as before (37).

For monitoring hyphal growth by live cell imaging, overnight cultures grown in YPD were diluted to OD_600_ of 0.1 into 200 μL BMDM, 2% CAS, or M199 medium with or without 10 mM crotonate in 96-well plates, and incubated at 37°C. Images were taken every 15 min for 5 h on a Leica DMi8 live cell imaging microscope at ×20.

### Hyphal filamentation on plates

To observe hyphal filamentation of colonies on solid agar media, *C. albicans* was grown overnight for 16 h at 30°C to stationary phase. The culture was diluted to give a cell suspension of around 20 cells/100 µL, and then evenly spread on plates using glass beads. Plates were then incubated for 37°C for 7 days. RPMI and M199 agar were prepared as 2× solutions of previously described constituents, filter sterilized, and added to autoclaved 2× agar, and CAS agar was autoclaved containing all described ingredients. Images of single colonies were taken on the Olympus MVX19 microscope at 0.63X.

### Western blots

For macrophage Westerns, whole cell lysates were prepared by boiling BMDM cell pellets in 2× Laemmli sample buffer for 5 min. Histone lysine acylations were analyzed as we have described before (16) using the following antibodies: anti-pan-KCr (PTM-Biolabs 501, 1:1,000 dilution in Tris-buffered saline with Tween [TBST] with 5% skim milk), anti-H3K18Cr (Abcam; ab195475, 1:1,000 dilution in TBST with 5% BSA), anti-H3K18Ac (Abcam 1191), anti-H3 (Abcam 179, 1:10,000 dilution in SuperBlock; Thermo Scientific, 37537), and anti-rabbit IgG secondary antibody (1:20,000 dilution in TBST with 5% skim milk). Detection was done with Amersham ECL reagent (Sigma, RPN2209). Our protocol for Western blot analysis of IL-1β is described in reference 43. For the experiments in this study, infection with *C. albicans* was at an MOI of 6. Samples were collected at 3, 6, and 9 h after infection. Western blots were done using the anti-mouse IL-1β antibody (R&D Systems; AF-401-NA, 1:1,000 dilution) and anti-goat HRP secondary antibody (1:10,000 dilution) and were detected with ECL reagent (SuperSignal West Dura Extended Duration Substrate, Thermo Fisher 34075). Actin was the loading control (anti-actin, Millipore MAB150, 1:5,000 dilution; anti-mouse IgG secondary antibody, 1:20,000 dilution). Lysate membranes were also stained with Ponceau S for total protein detection.

For *Candida*, whole cell protein extraction and Western blots were performed using our protocols described in reference 16, with minor adjustments. To assay Nrg1-MYC protein levels, overnight cultures of *C. albicans* (YPD at 30°C) were diluted to OD_600_ of 0.2 into YPD media as a yeast morphology control, or M199 media (pH 5.4) to induce hyphal growth, with or without 10 mM crotonate. Cells were harvested after 5 h at 37°C. To assay H3K9Cr, H3K9Ac, and H3, *C. albicans* cultures were grown in M199 media for 2 h with or without 10 mM crotonate and 1 µM trichostatin A, alongside a dimethyl sulfoxide (DMSO) vehicle control. Whole cell extracts were prepared and samples loaded on a 10% SDS-PAGE gel (Nrg1-MYC) or 12% SDS-PAGE gel (H3, H3K9Ac, and H3K9Cr). Primary antibodies were as follows: anti-actin (Millipore MAB150) and anti-MYC (Sigma, M4439) at a 1:5,000 dilution; H3K9Cr (Sigma, SAB5600124), H3K9Ac (Millipore, 07352) and H3 (Abcam, ab1791) at 1:1,000, 1:5,000, and 1:5,000 dilutions respectively. Secondary antibodies were as follows: HRP conjugated secondary anti-mouse (1:10,000 dilution) and HRP conjugated secondary anti-rabbit (1:20,000 dilution for H3K9Ac and H3 Westerns and 1:10,000 dilution for H3K9Cr). For the dot blot to test antibody specificity (Fig. S8), 5 μL of 5.0, 0.5, or 0.05 µg of acylated peptides was used, and the protocol of Gowans et al. was followed (12). ImageQuant was used for quantification of Western blot data.

### Seahorse experiments

Seeding of BMDMs was done in XF24 microplates with or without 10 mM crotonate or acetate (2 × 10^5^ cells/well). The metabolic activity of *Candida* was determined by adding 2 × 10^5^ cells/well of *Candida* treated with or with 10 mM crotonate or acetate for 1 h prior to the assay. Seahorse XF24 and XF Glycolysis Stress Test Kit User Guide were used to determine the OCR and ECAR, as we described previously (37).

### Dual *Candida*-macrophage RNAseq analysis

For RNAseq experiments, BMDMs were primed with LPS (50 ng/mL) for 3 h, followed by infection with *C. albicans* SC5314 (MOI 6), and sample collection at 1 and 3 h post-phagocytosis. Three independent experiments were performed. Total RNA isolation and library preparation by the PAT-seq method has been described in detail by our lab (37). The *C. albicans* reference genome was SC5314 Assembly 21; for mouse, it was Mm39 assembly (Ensembl 107); alignments were performed separately using the STAR aligner tool (70). Genes with at least 10 reads in at least three samples were used for analysis. For differentially expressed genes, a cutoff of ≥1.5 fold change with <0.05 FDR was used.

### Chromatin immunoprecipitation

Chromatin immunoprecipitation was performed as described in reference 49 with modifications.

Cells were grown for 3 h and cross-linked by adding formaldehyde to a final concentration of 1%. Fixed cultures were shaken for 10min at 30°C on an orbital shaker at 100rpm. Then 4.5 M Tris, pH 8, was added to a final concentration of 750 mM, shaking for 5 min under the same conditions. Cells were pelleted at 3,000 rpm for 5 min at 4°C, washed twice with TBS, and resuspended in 400 µL of lysis buffer 1 (50 mM HEPES-KOH, pH 7.5, 150 mM NaCl, 1 mM EDTA, 1% Triton X-100, 0.1% sodium deoxycholate, and 0.1% SDS) containing protease inhibitor cocktail (Roche). Cells were lysed using FastPrep-24 Classic bead beating lysis system. Chromatin was sheared to 200–700 bp by sonication using a Bioruptor diagenode (at 4°C) over eight cycles for 20 seconds on high power and 20 seconds off.

For IP, 2.5 µg of fixed chromatin was added to 2 µg anti-Myc antibody (Sigma, M4439) and lysis buffer to a final volume of 500 µL. The IP was incubated overnight at 4°C before addition of 20 µL of 50% suspension of protein A Sepharose beads (Abcam, ab193256) in lysis buffer and was incubated for 2 h at 4°C with shaking. The beads were pelleted and, following discarding of the supernatant, were washed with several buffers for 5 min in sequence. Firstly, twice with lysis buffer 1, then twice with lysis buffer 2 (50 mM HEPES-KOH, pH7.5, 500 mM NaCl, 1 mM EDTA, 1% Triton X-100, 0.1% sodium deoxycholate, and 0.1% SDS), twice in wash buffer (10 mM Tris-HCl, pH 8, 0.25 M LiCl, 1 mM EDTA, 0.5% NP-40, and 0.5% sodium deoxycholate), and finally once in Tris-EDTA (TE) (10 mM Tris-HCl, pH 8, 1 mM EDTA). Subsequently, 75 µL elution buffer (50 mM Tris-HCl, pH 7.5, 10 mM EDTA, and 1% SDS) was added to every sample, and they were incubated for 10 min at 65°C. Samples were centrifuged at 3,000 rpm for 1 min, and the eluate was retained. Beads were rewashed with another 75 µL elution buffer, and the eluates were combined.

For input control, 20 µL input chromatin was added to 180 µL of elution buffer, and both IP and input samples were incubated overnight at 65°C to reverse crosslinks. One hundred fifty microliters of proteinase K solution (TE, 60 µg/mL glycogen and 500 µg/mL proteinase K) was added to each sample and incubated for 2 h at 37°C. DNA was cleaned using the phenol chloroform method with phenol/chloroform/isoamyl alcohol solution (25:24:1) followed by an additional chloroform step. Fifteen microliters 5 M NaCl and 800 µL ice cold 100% ethanol were added and DNA allowed to precipitate at −20°C for 20 min. Samples were then pelleted at 14,000 rpm at 4°C for 10 min, washed with 70% ice cold ethanol and allowed to air dry. Samples were resuspended in 40 µL nuclease-free water. Both input and chromatin immunoprecipitation sample DNA concentrations were determined using Nanodrop, normalized, and analyzed by quantitative PCR (qPCR).

### Quantitative PCR

qPCR analysis of gene expression was performed as previously described (16), with minor adjustments. Primers are shown in Table S2. For analysis of the effect of crotonate on hyphal-specific genes, overnight-grown cultures were diluted to OD_600_ of 0.3 in YPD (yeast) or M199 (hyphae) and grown for 5 h at 30°C (yeast) or 37°C (hyphae). For analysis of cells treated with a combination of crotonate plus TSA, cells were grown on plates for 7 days at 37°C; individual colonies were extracted from the agar and processed as normal. *RDN25* was used for normalization. Data were produced by the Roche LightCycler 480 system and analyzed by LinReg software. Statistical analysis was done on the normalized values using GraphPad Prism.

## Data Availability

Data were deposited in GEO under accession number GSE234554.

## SUPPLEMENTAL MATERIAL

Dataset S1 mbio.02605-23-s0001.xlsx RNAseq data analyses.

Dataset S2 mbio.02605-23-s0002.xlsx Data used to construct graphs in figures.

Supplemental figures mbio.02605-23-s0003.pdf Fig. S1 to S9.

Supplemental legends mbio.02605-23-s0004.docx Legends for supplemental movies and data sets.

Table S1 mbio.02605-23-s0005.pdf Strains used in the study.

Table S2 mbio.02605-23-s0006.pdf Primers used in the study.

Movie S1 mbio.02605-23-s0007.mp4 Live cell imaging of *C. albicans* growing in RPMI + serum medium in the presence or absence of crotonate.

Movie S2 mbio.02605-23-s0008.mp4 Live cell imaging of *C. albicans* growing in medium M199 in the presence or absence of crotonate.

Movie S3 mbio.02605-23-s0009.mp4 Live cell imaging of *C. albicans* growing in CAS medium in the presence or absence of crotonate.

ASM does not own the copyrights to Supplemental Material that may be linked to, or accessed through, an article. The authors have granted ASM a non-exclusive, world-wide license to publish the Supplemental Material files. Please contact the corresponding author directly for reuse.
