# Supplementary material for: The short-chain fatty acid crotonate reduces invasive growth and immune escape of Candida albicans by regulating hyphal gene expression
Source: mBio. 2023 Nov 6;14(6):e02605-23. doi: 10.1128/mbio.02605-23 (PMC10746253; doi:10.1128/mbio.02605-23)
Supplement: Supplemental figures — Fig. S1 to S9. [file mbio.02605-23-s0003.pdf]

Figure S1

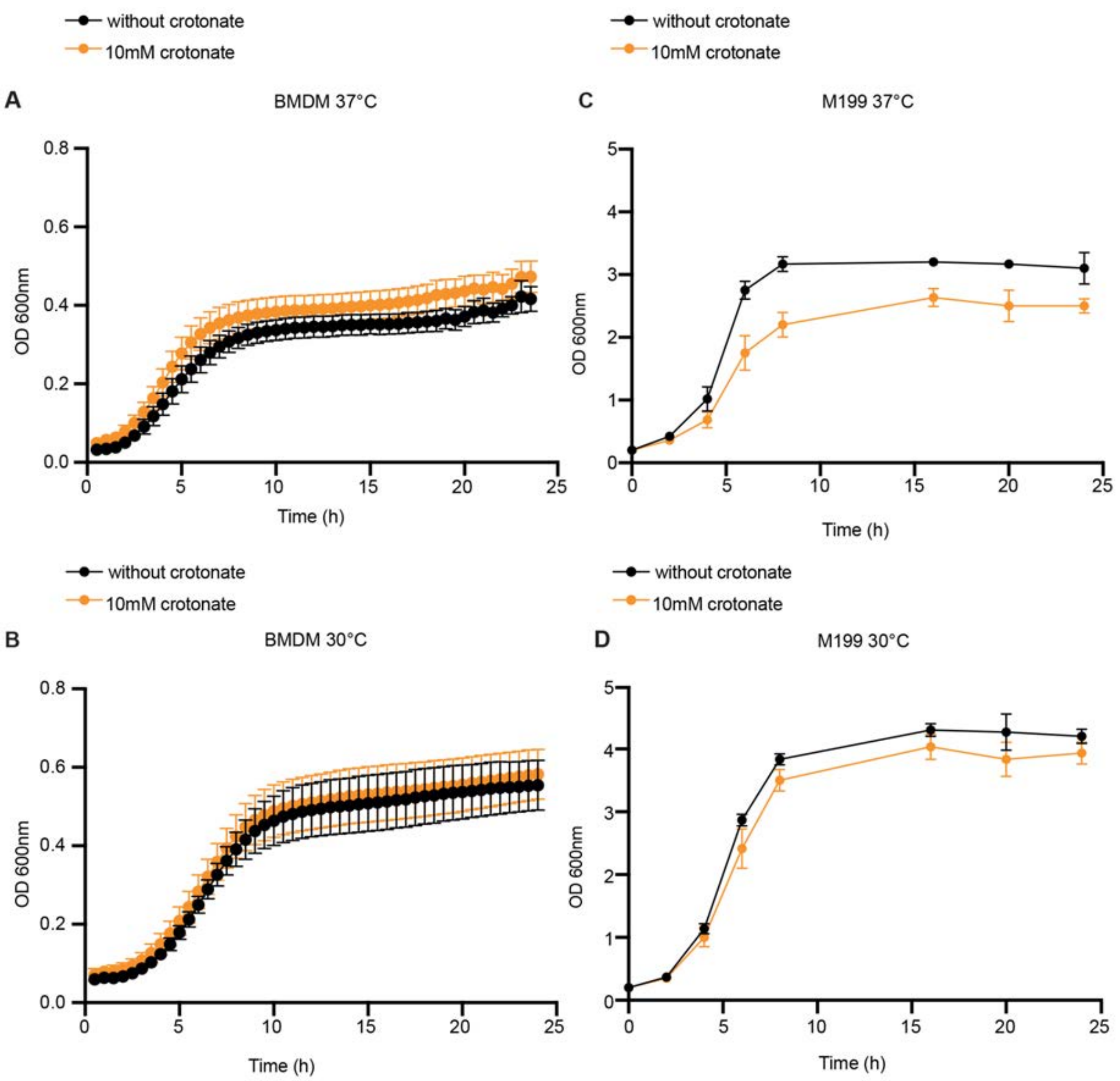

Figure S1. Growth of *C. albicans* in the presence of crotonate

- **A.** Growth curves of C. albicans in the presence of crotonate in macrophage infection medium (BMDM, see Materials and methods for its composition) at 37 °C. Shown are the average and SEM of 3 independent experiments.
- **B.** As in A but cultures were grown in BMDM medium 30 °C
- **C.** As in A but cultures were grown in M199 medium at 37 °C.
- **D.** As in A but in M199 medium at 30 °C.

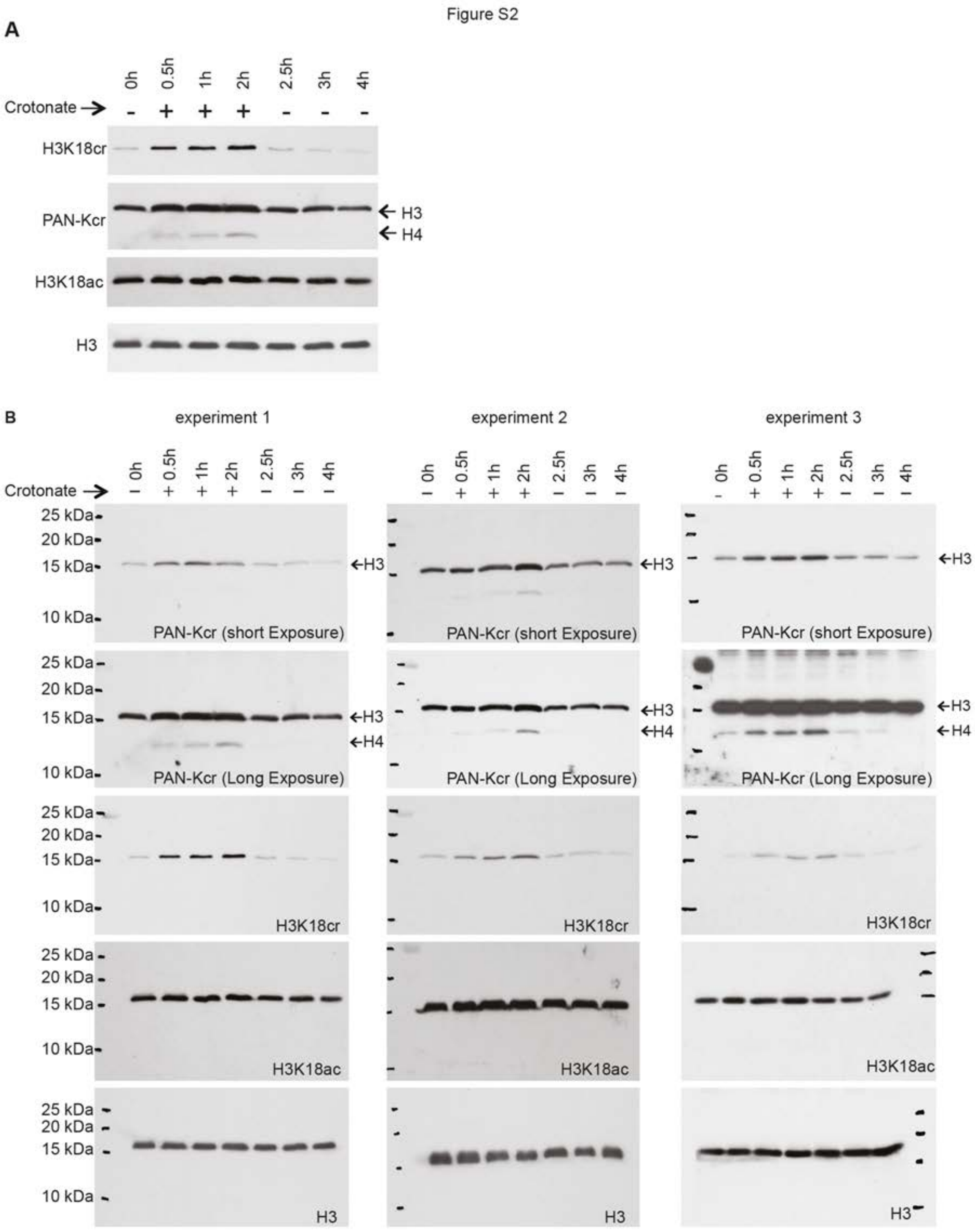

Figure S2. Crotonate dynamically regulates histone croyonylation in macrophages

A. Western blot of lysine crotonylation in BMDMs following 10 mM crotonate treatment. Samples were taken at the indicated time points. For crotonate removal, BMDM culture media with crotonate was removed, washed with PBS, and supplemented with fresh media without crotonate (indicated by - and starting at the 2.5 h time point). Individual gels (x4) with identical whole-cell extracts were loaded and Western blots performed

with antibodies to detect H3K18cr, total lysine crotonylation (Pan-Kcr), H3K18ac, and total H3, respectively. A representative experiment is shown here.

B. Uncropped western blots of the experiment in A and two other independent biological repeats.

Figure S3

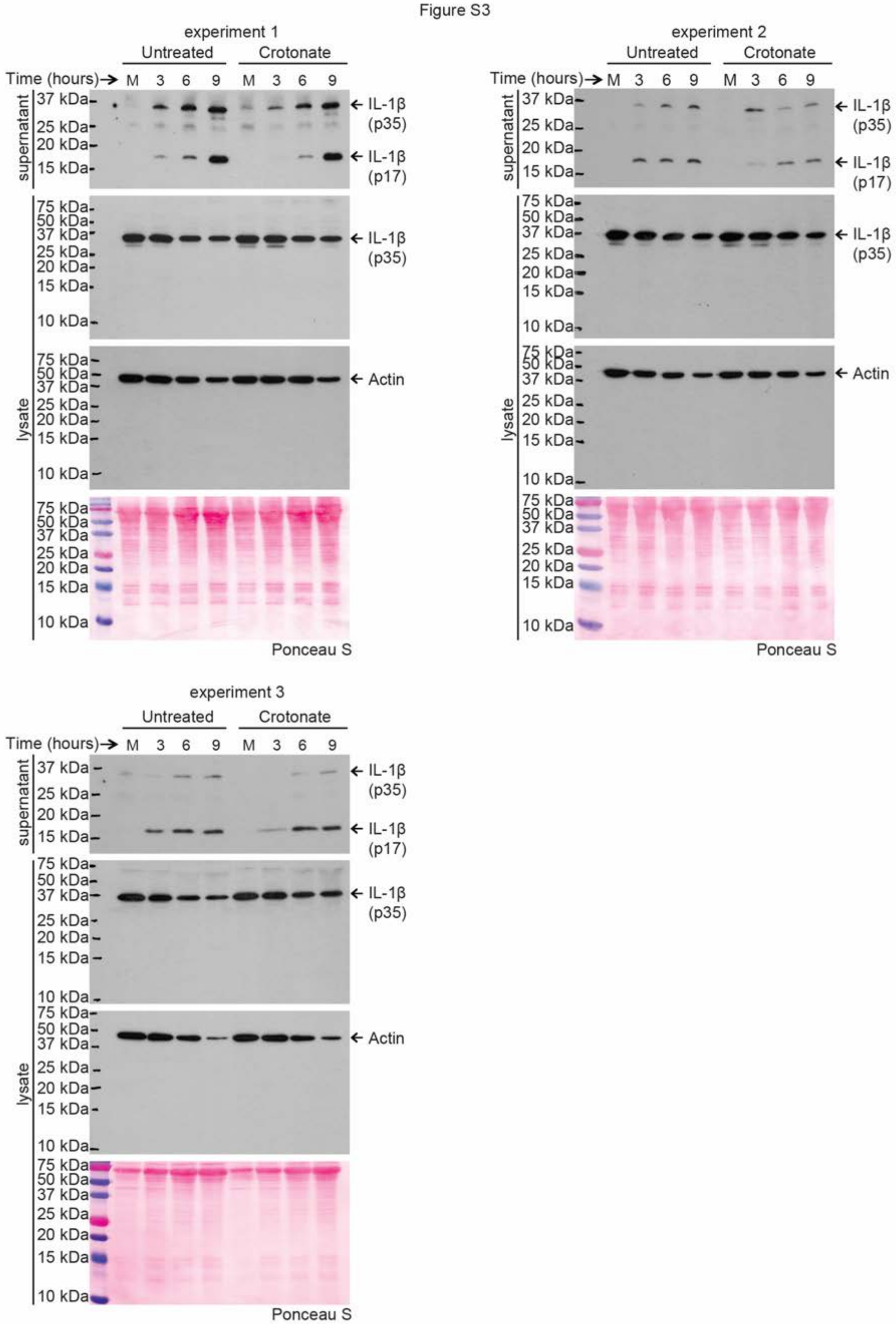

experiment 1
Untreated
Crotonate
Time (hours)
M 3 6 9 M 3 6 9
supernatant
37 kDa
25 kDa
20 kDa
15 kDa
IL-1β (p35)
IL-1β (p17)
75 kDa
50 kDa
37 kDa
25 kDa
20 kDa
15 kDa
10 kDa
IL-1β (p35)
Actin
lysate
Ponceau S
experiment 2
Untreated
Crotonate
Time (hours)
M 3 6 9 M 3 6 9
supernatant
IL-1β (p35)
IL-1β (p17)
IL-1β (p35)
Actin
lysate
Ponceau S
experiment 3
Untreated
Crotonate
Time (hours)
M 3 6 9 M 3 6 9
supernatant
IL-1β (p35)
IL-1β (p17)
IL-1β (p35)
Actin
lysate
Ponceau S

Figure S3. Uncropped IL-1ß Western blots

Shown here are 3 independent biological repeats of the experiment described in Figure 1F. Pounceau-stained membranes are shown as a loading control.

Figure S4

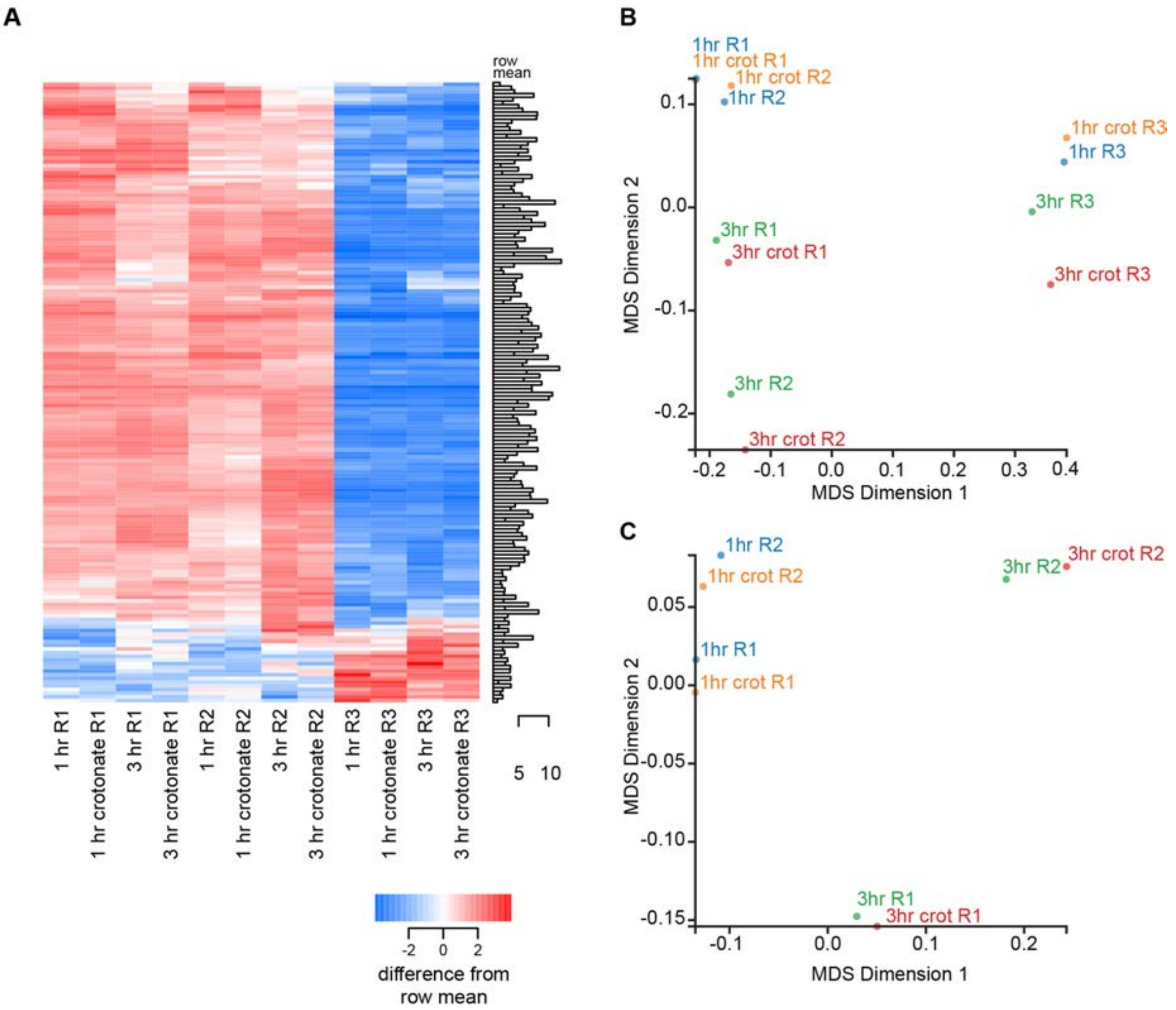

Figure S4. Macrophage RNAseq data in response to crotonate

A. Heatmap of macrophage genes showing at least 20-fold change difference relative to row mean.

B. Multidimensional Scaling plot of the macrophage RNAseq data with all three repeats.

C. Multidimensional Scaling plot of the macrophage RNAseq data without repeat 3.

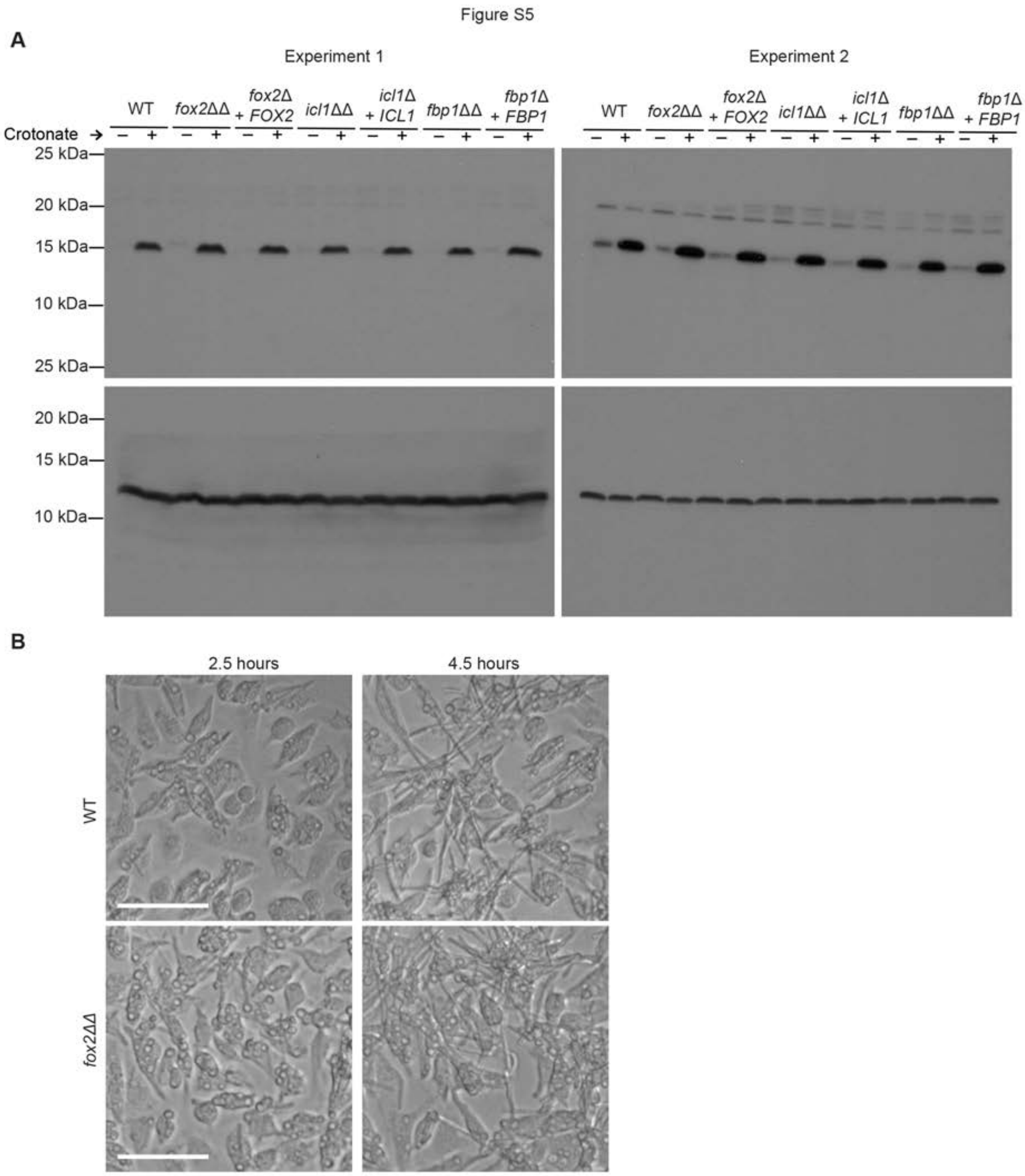

Figure S5. Morphogenesis of the β-oxidation mutant of *C. albicans* within macrophages and Western blots of metabolic mutants in response to crotonate

A. Shown here are uncropped blots of the experiment described in Figure 3E and an additional independent experiment.

B. Macrophages (BMDMs) were infected with the indicated strains (MOI 6). Images are from the live cell imaging shown in Figure 3F, at 4.5 h post-infection. Scale bar is 50 μm.

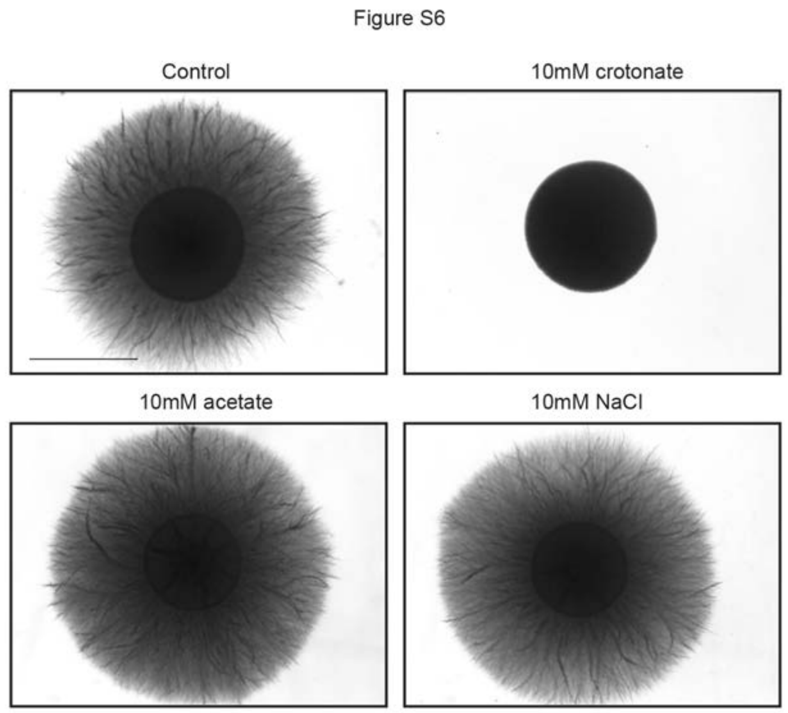

Figure S6. Control experiments for the effects of crotonate on hyphal formation on M199 plates.

Fungal cultures were spread for single colonies on the indicated plates and photographed after 7 days of growth at 37 °C. This control experiment was performed once. Scale bar is 4mm.

Figure S7

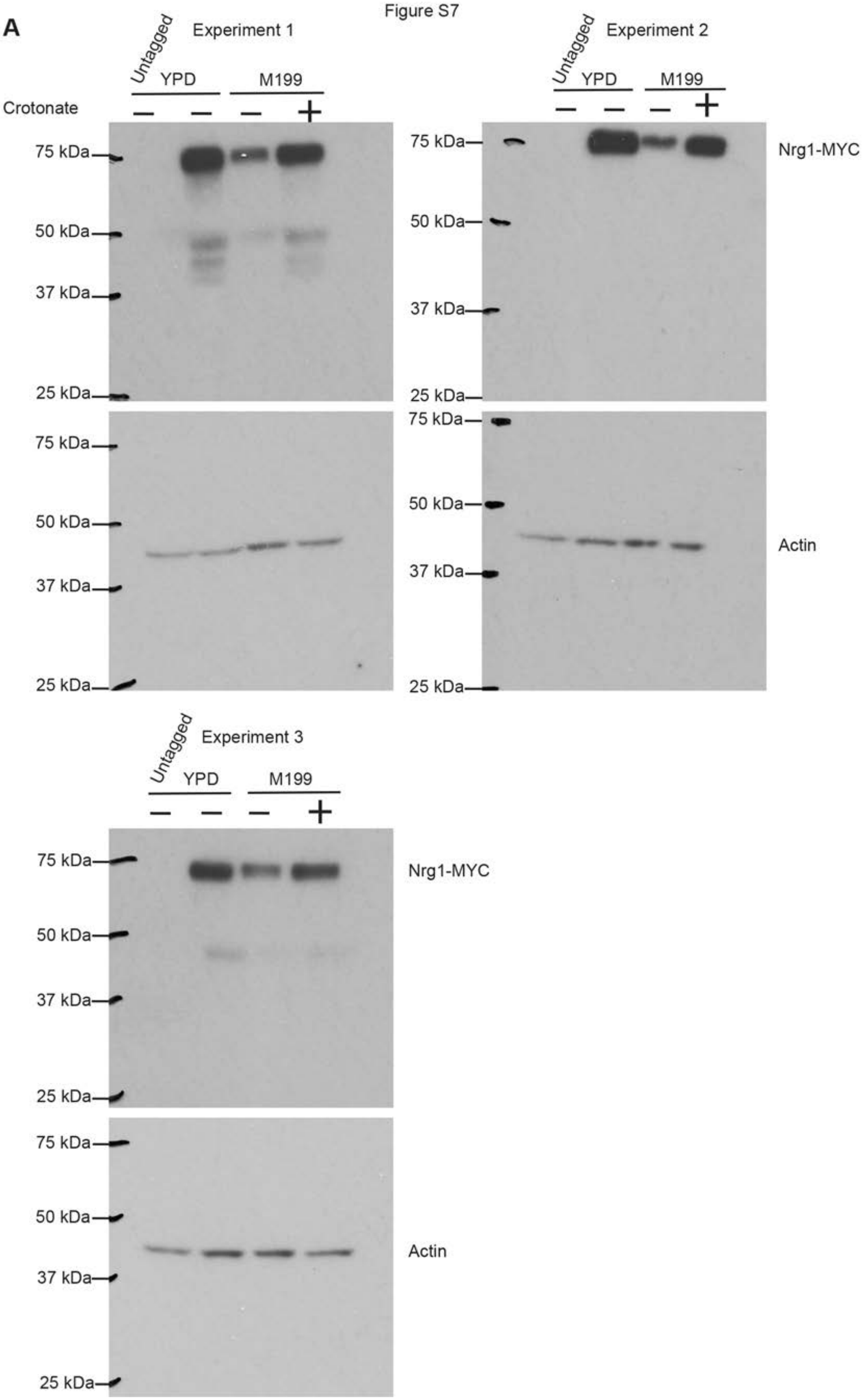
A
Experiment 1
Untagged
YPD
M199
Crotonate
– – – +
75 kDa
50 kDa
37 kDa
25 kDa
Experiment 2
Untagged
YPD
M199
– – – +
Nrg1-MYC
Actin
Experiment 3
Untagged
YPD
M199
– – – +
Nrg1-MYC
Actin

Figure S7. Western blots of Nrg1-Myc in response to crotonate

Shown here are uncropped blots of the experiment described in Figure 5A and two other independent biological repeats.

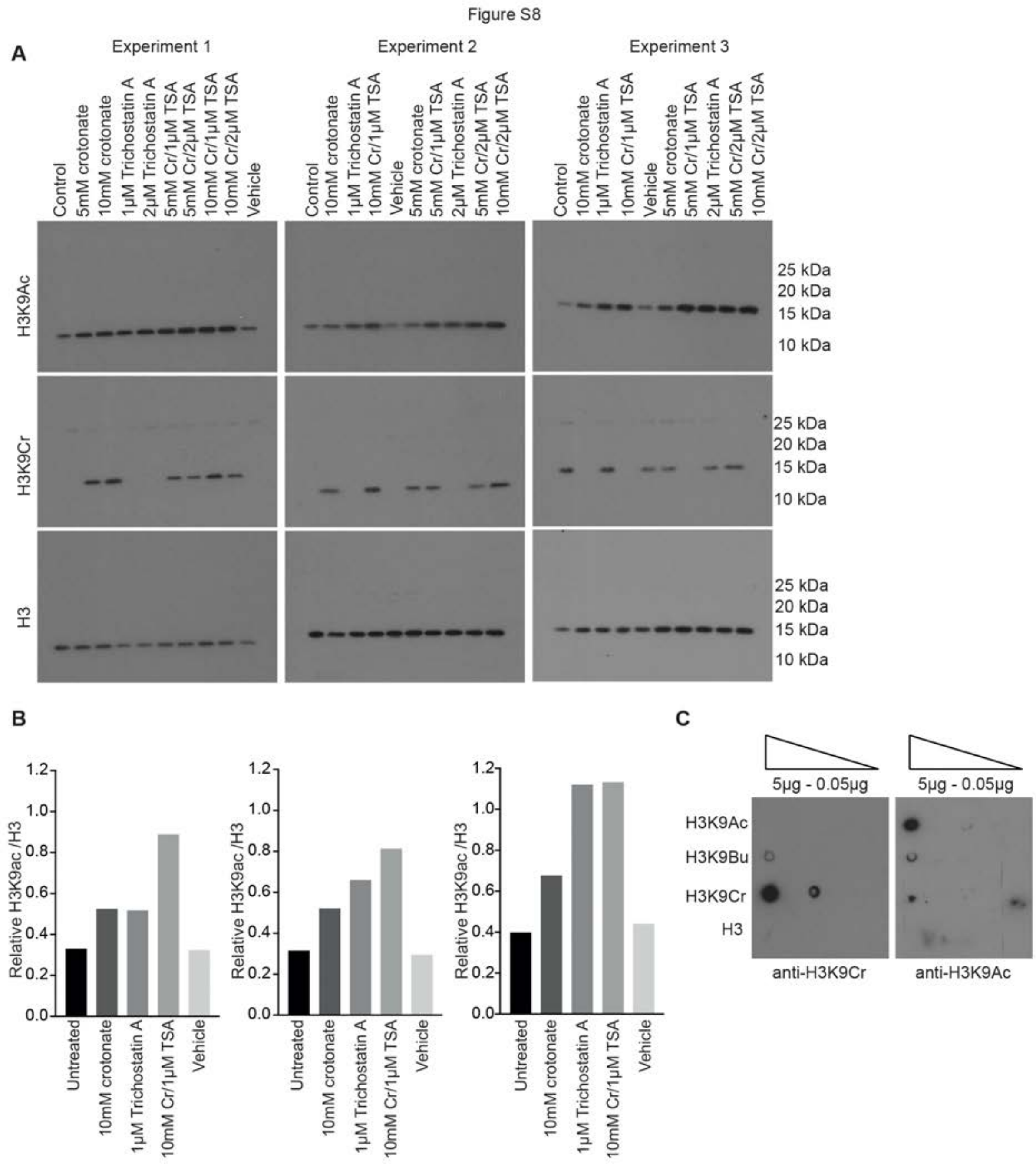

Figure S8. Western blots of histone acylations in response to crotonate and TSA

- **A.** Shown here are uncropped blots of the experiment described in Figure 6A and two other independent biological repeats
- **B.** Shown here are quantification of western blots shown in panel A. These same data are shown together in Figure 6B.
- **C.** Dot blot assays testing the specificity for the anti-H3K9cr an anti-H3K9ac antibody.

Figure S9

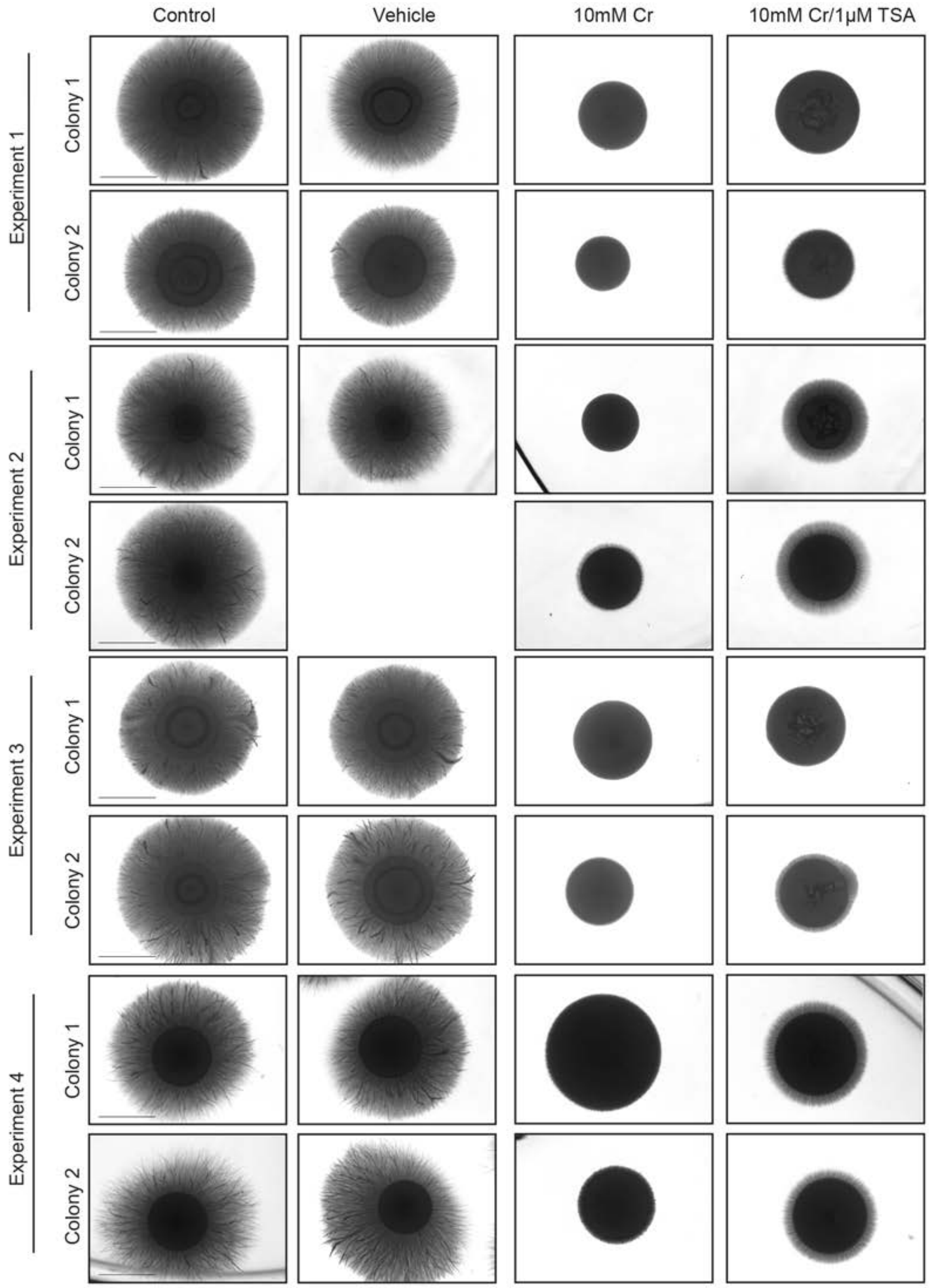

Figure S9. Rescue of crotonate's hyphal inhibition by the histone deacetylase inhibitor TSA

The experiments were done as described in Figure 6C. Hyphal morphogenesis on M199 plates is shown +/- 10 mM crotonate and/or 1 μM TSA. Plates were photographed after 7 days of growth at 37 °C. Scale bar is 4 mm.
