# Supplementary material for: The short-chain fatty acid crotonate reduces invasive growth and immune escape of Candida albicans by regulating hyphal gene expression
Source: mBio. 2023 Nov 6;14(6):e02605-23. doi: 10.1128/mbio.02605-23 (PMC10746253; doi:10.1128/mbio.02605-23)
Supplement: Supplemental legends — Legends for supplemental movies and data sets. [file mbio.02605-23-s0004.docx]

**Movie S1. Live cell imaging of *C. albicans* growing in RPMI + serum medium in the presence or absence of crotonate.** Movies were collected as described in Figure 4 and methods. Videos were scaled and time-stamped in Fiji and merged using Adobe Premiere Pro.

**Movie S2. Live cell imaging of *C. albicans* growing in medium M199 in the presence or absence of crotonate.** Movies were collected as described in Figure 4 and methods. Videos were scaled and time-stamped in Fiji and merged using Adobe Premiere Pro.

**Movie S3. Live cell imaging of *C. albicans* growing in CAS medium in the presence or absence of crotonate.** Movies were collected as described in Figure 4 and methods. Videos were scaled and timestamped in Fiji and merged using Adobe Premiere Pro.

**Dataset S1. RNAseq analysis of crotonate-induced changes to the *C. albicans* transcriptomes during macrophage infections.**

**Dataset S2. Data used to prepare the graphs shown in the figures.**
