# Supplementary material for: The short-chain fatty acid crotonate reduces invasive growth and immune escape of Candida albicans by regulating hyphal gene expression
Source: mBio. 2023 Nov 6;14(6):e02605-23. doi: 10.1128/mbio.02605-23 (PMC10746253; doi:10.1128/mbio.02605-23)
Supplement: Table S1 — Strains used in the study. [file mbio.02605-23-s0005.pdf]

Table S1. Strains used in this study.

Identifier	Short name	Strain Genotype	Background	Source
YCAT229	SC5314	Wild Type SC5314 - prototroph	SC5314	Fonzi and Irwin, 1993
YCAT639	SN250	*ura3Δ::λimm434::URA3-IRO1/ura3Δ::λimm434 arg4::hisG/arg4::hisG his1::hisG/his1::hisG leu2::hisG::CdHIS1/leu2::hisG::CmLEU2*	SN152	Noble et al. 2010; Fungal Genetics Stock Centre
YCAT1050	*nrg1ΔΔ*	*nrg1Δ::C.m.LEU2/nrg1Δ::C.d.HIS1*	SN152	Homann et al. 2009; Transcription factor deletion library
YCAT754	*tup1ΔΔ*	*tup1::LEU2/tup1::HIS1*	SN152	Homann et al. 2009; Transcription factor deletion library
YCAT1052	Nrg1-Myc	ura3Δ::λimm434/ura3Δ::λimm434 NRG1/NRG1-13MYCFLAG-URA3 Clone1	CAI4	Lu et al. 2011
YCAT1223	*fox2ΔΔ*	ura3:: λimm434/ura3:: λimm434 fox2::hisG/ fox2::hisG RPS10/rps10::URA3	CAI4-F2	Ramirez and Lorenz, 2007
YCAT1224	*fox2Δ+FOX2*	ura3:: λimm434/ura3:: λimm434 fox2::hisG/ fox2::hisG RPS10/rps10::FOX2-URA	CAI4-F2	Ramirez and Lorenz, 2007
YCAT1225	*icl1ΔΔ*	*ura3:: λimm434/ura3:: λimm434 icl1::hisG/ icl1::hisG RPS10/rps10::URA3*	CAI4-F2	Ramirez and Lorenz, 2007
YCAT1226	*icl1Δ+ICL1*	ura3:: λimm434/ura3:: λimm434 icl1::hisG/ icl1::hisG RPS10/rps10::ICL1-URA3	CAI4-F2	Ramirez and Lorenz, 2007
YCAT1227	*fbp1ΔΔ*	*ura3:: λimm434/ura3:: λimm434 fbp1::hisG/ fbp1::hisG RPS10/rps10::URA3*	CAI4-F2	Ramirez and Lorenz, 2007

Table S1. Strains used in this study.

YCAT1228	*fbp1Δ+FBP1*	*ura3:: λimm434/ura3:: λimm434 fbp1::hisG/ fbp1::hisG RPS10/rps10::FBP-URA3*	CAI4-F2	Ramirez and Lorenz, 2007
YCAT822	TetR control (PCY87)	*ade2::hisG/ade2::hisG ura3::imm434/ura3::imm434 ENO1/eno1::ENO1-tetR-ScHAP4AD-33HA-ADE2, URA3*	CAI8	Carlisle et al. 2009
YCAT821	*tetO-UME6 (MBY38)*	*ade2::hisG/ade2::hisG ura3::imm434/ura3::imm434 ENO1/eno1::ENO1-tetR-ScHAP4AD-33HA-ADE2, URA3-tetO-UME6*	CAI8	Carlisle et al. 2009
