# Supplementary material for: The short-chain fatty acid crotonate reduces invasive growth and immune escape of Candida albicans by regulating hyphal gene expression
Source: mBio. 2023 Nov 6;14(6):e02605-23. doi: 10.1128/mbio.02605-23 (PMC10746253; doi:10.1128/mbio.02605-23)
Supplement: Table S2 — Primers used in the study. [file mbio.02605-23-s0006.pdf]

## Table S2. Primers used in this study.

| **qPCR primers** | | | |
|---|---|---|---|
| **Identifier** | **Name** | **Purpose** | **Sequence** |
| qC11 | *ECE1* Fw | For qPCR of *ECE1* | TGCCGTCGTCAGATTGCCAGA |
| qC12 | *ECE1* Rev | For qPCR of *ECE1* | AGGCCAACATCTGGAACGCCA |
| qC294 | *RDN25* Fw | For qPCR of *RDN25* | GAGAGGAACCGTTCATTCAGATAAT |
| qC295 | *RDN25* Rev | For qPCR of *RDN25* | CTATGGTCCAGCGACTAAAAAGTCT |
| qC320 | *HWP1* Fw | For qPCR of *HWP1* | CTGCTCCTGAAATGACTCCAGCTG |
| qC321 | *HWP1* Rev | For qPCR of *HWP1* | GAGACGACAGCACTAGATTCCGGA |
| qC519 | *ALS3 Fw* | For qPCR of *ALS3* | AATGGTGGTGGTAATGCTCCA |
| qC520 | *ALS3 Rev* | For qPCR of *ALS3* | TTGCGATTGAGATTGGTTGG |
| qC482 | *ACT1* Fw | For ChIP qPCr | CCCAGGTATTGCTGAACGTA |
| qC483 | *ACT1* Rev | For ChIP qPCr | GAACCACCAATCCAGACAGA |
| qC521 | *ALS3* Fw | For ChIP qPCr | GGGAGCATCATTAAGAATTAGACGA |
| qC530 | *ALS3* Rev | For ChIP qPCr | AAATGGCGGACTCAGGGTTT |
